# Cytomegalovirus and Epstein–Barr virus co-infected young and middle-aged adults can have an aging-related T-cell phenotype

**DOI:** 10.1038/s41598-023-37502-5

**Published:** 2023-07-05

**Authors:** Marloes I. Hofstee, Alper Cevirgel, Mary-Lène de Zeeuw-Brouwer, Lia de Rond, Fiona van der Klis, Anne-Marie Buisman

**Affiliations:** 1 Centre for Infectious Disease Control, National Institute for Public Health and the Environment (RIVM), Antonie Van Leeuwenhoeklaan 9, 3721 MA Bilthoven, The Netherlands; 2 Department of Medical Microbiology and Infection Prevention, Virology and Immunology Research Group, University Medical Center Groningen, Groningen, The Netherlands

**Keywords:** Herpes virus, T cells, Infection

## Abstract

Cytomegalovirus (CMV) is known to alter circulating effector memory or re-expressing CD45RA^+^ (TemRA) T-cell numbers, but whether Epstein–Barr virus (EBV) does the same or this is amplified during a CMV and EBV co-infection is unclear. Immune cell numbers in blood of children and young, middle-aged, and senior adults (*n* = 336) were determined with flow cytometry, and additional multivariate linear regression, intra-group correlation, and cluster analyses were performed. Compared to non-infected controls, CMV-seropositive individuals from all age groups had more immune cell variance, and CMV^+^ EBV^−^ senior adults had more late-differentiated CD4^+^ and CD8^+^ TemRA and CD4^+^ effector memory T-cells. EBV-seropositive children and young adults had a more equal immune cell composition than non-infected controls, and CMV^−^ EBV^+^ senior adults had more intermediate/late-differentiated CD4^+^ TemRA and effector memory T-cells than non-infected controls. CMV and EBV co-infected young and middle-aged adults with an elevated BMI and anti-CMV antibody levels had a similar immune cell composition as senior adults, and CMV^+^ EBV^+^ middle-aged adults had more late-differentiated CD8^+^ TemRA, effector memory, and HLA-DR^+^ CD38^−^ T-cells than CMV^+^ EBV^−^ controls. This study identified changes in T-cell numbers in CMV- or EBV-seropositive individuals and that some CMV and EBV co-infected young and middle-aged adults had an aging-related T-cell phenotype.

## Introduction

Cytomegalovirus (CMV) is a β-herpesvirus that persists lifelong in humans by residing within bone marrow resident CD34^+^ myeloid progenitor cells, peripheral blood CD14^+^ monocytes derived from these cells^1,2^, and endothelial cells^3^. Although for most individuals a persistent CMV infection does not result in any clinical symptoms, the immune cell composition in blood of these individuals has been altered dramatically, which can have implications on de novo T-cell responses^4^. For example, blood samples of CMV-seropositive individuals contain more CD4^+^ and CD8^+^ effector memory T-cells and effector memory T-cells that re-express CD45RA (TemRA)^5–7^. These effector memory and TemRA T-cells found in higher quantities in blood of CMV-seropositive people lack expression of CD27 and CD28 and, thus, have a terminally/late-differentiated phenotype, and can express the senescence markers CD57 and KLRG-1^5^. There are also some reports describing that CMV seropositivity affect numbers of B-cells^8,9^, NKT cells^10,11^, and NK CD56^dim^ cells in blood^12^. However, it should be kept in mind that aging has an impact on immune cell composition as well. This is generally referred to as immunosenescence and has been linked to NK cells becoming more cytolytic (CD56^dim^) than cytotoxic (CD56^bright^)^13^, and also has been associated to decreases in the number of DC cells^14^, B-cells^8,15^, and naïve T-cells in blood, while the number of central memory T-cells increase^16,17^.

Epstein–Barr virus (EBV) is another herpesvirus that can persist in humans for a life time. This γ-herpes virus infects primarily B-cells, but also epithelial cells^18^. Potentially, EBV alters T-cell numbers in blood in a similar fashion as CMV, but to date only a few reports have sought to clarify this^19–22^. Studying EBV-related effects on immune cell numbers can be challenging given that the prevalence of the virus in adults is high and for some study cohorts is even above 90%^5,7^. One study reported that EBV-seropositive individuals have especially early-differentiated CD45RA^+/−^ EBV-specific CD8^+^ T-cells^19^ and another study showed that intermediate-differentiated CD45RA^+/−^ EBV-specific CD8^+^ T-cells expand in EBV-seropositive individuals, but in a lower magnitude when the individuals were also CMV-seropositive^20^. In children, a recent study described that an EBV latent infection led to especially more late-differentiated CD8^+^ effector memory T-cells compared to non-infected children and this increase was even more pronounced in children having a CMV and EBV co-infection^21^. Potentially, a CMV and EBV co-infection might result in a more altered immune cell composition in blood than a CMV- or EBV-mono-infection. Although, to the best of our knowledge, this has not been studied in great detail yet.

In this study, we explored the impact of having a CMV mono-infection, EBV mono-infection, and a co-infection with CMV and EBV on the immune cell composition in blood of individuals from different age groups, with a specific focus on cells of the T-cell compartment. The study population consisted of children, young adults, middle-aged adults, and senior adults, respectively 4–8 year, 18–25 year, 39–45 year, and 64–70 year of age. CMV- and EBV-seropositive individuals were identified by measuring antibody levels in plasma or serum against these viruses, as these antibodies remain present throughout the latent infection. With extensive flow cytometric analysis, which included innate and adaptive immune cell markers, the immune cell composition in blood was mapped for the study participants, whom were grouped based on age, CMV seropositivity, and/or EBV seropositivity. Additionally, a multivariate correlation was performed to assess whether age, CMV-, EBV-antibody levels, or sex could be correlated with these immune cell quantities. Since multifactorial aspects have influenced the immune subset variance between individuals during life, we investigated the study participants based on their immune profiles alone by utilizing a clustering approach, a method which has been described recently^23^. Previously, this clustering analysis identified immune subset that are influenced by age and/or CMV seropositivity^23^. In this study, the cluster analysis will be performed to investigate whether a CMV and EBV co-infection causes an individual to have a more altered immune cell composition than a person with a CMV mono-infection. Overall, the results of this study will give more insight into alterations in immune cell composition in blood of CMV-seropositive and/or EBV-seropositive individuals.

## Results

### Demographics of the study population

The demographics of the study population are enlisted in Table 1 and consisted of 336 individuals. These individuals were subdivided into four different age groups; 4–8 year old children (*n* = 62), 18–25 year old young adults (*n* = 89), 39–45 year old middle-aged adults (*n* = 93), and 64–70 year senior adults (*n* = 92). The percentage of individuals with an unhealthy BMI and diseases that have been hypothesized to potentially be CMV-related increased with age and was for senior adults 71.1% (compared to children and young adults: *p* < 0.001 and compared to middle-aged adults: *p* = 0.002) and 10.9% (compared younger age groups: *p* < 0.001), respectively. Eighteen percent of young adults smoked. Children had the highest score for acute clinical symptoms (43%) and senior adults had the lowest (22.1%), but these results were not significantly different.

**Table 1:** Demographics and CMV- and EBV-seroprevalence of the study population.

	Children 4–8 years	Young adults 18–25 years	Middle-aged adults 39–45 years	Senior adults 64–70 years
*Characteristics*				
Number (%)	62 (18.5%)	89 (26.5%)	93 (27.7%)	92 (27.4%)
Age (mean ± SD)	6.1 ± 1.3	21.6 ± 2.2	42 ± 1.7	67 ± 1.7
Sex (% women)	28 (45%)	61 (69%)*^a^	59 (63%)	52 (57%)
BMI (mean ± SD)	14.9 ± 1.6	23 ± 3.9	25.4 ± 4.8	26.3 ± 7.3
Unhealthy BMI (%)	11 (21.6%)	20 (24.4%)	34 (43.6%)	64 (71.1%)***^a,b,^**^c^
Smoking (% yes)	0 (0%)	16 (18%)	7 (7.5%)	12 (13%)
Score risk diseases (%)†	1 (0.4%)	4 (1.1%)	6 (1.6%)	40 (10.9%)***^a,b,c^
Score acute clinical symptoms (%)†	71 (43%)	74 (33.3%)	76 (33.8%)	45 (22.1%)
*Seroprevalence (number (%))*				
CMV^+^	16 (25.8%)**^d^	22 (24.7%)*^c,^***^d^	42 (45.2%)*^b^	52 (56.5%)**^a,^***^b^
EBV^+^	25 (40.3%)***^b,c,d^	66 (74.2%)***^a^	76 (81.7%)***^a^	70 (76.1%)***^a^
CMV^−^ EBV^−^	26 (41.9%)**^b,^***^c,d^	17 (19.1%)*^d^	10 (10.8%)	5 (5.4%)
CMV^−^ EBV^+^	18 (29%)	49 (55.1%)*^a^	40 (43%)	34 (37%)
CMV^+^ EBV^−^	8 (12.9%)	5 (5.6%)	6 (6.5%)	15 (16.3%)
CMV^+^ EBV^+^	7 (11.3%)	17 (19.1%)	36 (38.7%)**^a,^*^b^	36 (39.1%)**^a,^*^b^
No measurement or inconclusive EBV serostatus	3 (4.8%)	1 (1.1%)	1 (1.1%)	2 (2.2%)

Data in bold was significantly higher and data in bold and underlined was significantly lower. In superscript it is indicated whether the value was significantly different from the 4–8 years (a), 18–25 years (b), 39–45 years (c), or 64–70 years (d) age group. * *p* < 0.05, ** *p* < 0.01, and *** *p* < 0.001. †Reported numbers are the sum of the weighted scores and the percentages are the percentage from the highest possible score.

The percentage of CMV-seropositive children was 25.8%, and 24.7% of the young adults was CMV-seropositive. CMV seropositivity increased significantly with age and was 45.2% for the middle-aged adults (compared to young adults: *p* = 0.027) and 56.5% for the senior adults (compared to children and young adults: *p* = 0.001 and *p* < 0.001, respectively). EBV seropositivity or CMV and EBV seropositivity, respectively, was for the children 40.3% (significantly lower than the older age groups: *p* < 0.001) and 11.3%, for the young adults 74.2% and 19.1%, for the middle-aged adults 81.7% and 38.7% (significantly higher than children and young adults: *p* = 0.002 and *p* = 0.025, respectively), and for the senior adults 76.1% and 39.1% (significantly higher than children and young adults: *p* = 0.001 and *p* = 0.018, respectively).

With flow cytometric analysis, the absolute cell numbers in blood of different T-cell subsets, B-cells, NK-cells, NKT cells, granulocytes, and monocytes for these study participants were determined (Supplementary Table S1). From this initial analysis without taking into account CMV or EBV seropositivity, it appeared that some immune cell numbers declined with age e.g., CD4^+^ and CD8^+^ naïve T-cells, B-cells, and CD56^Bright^ NK-cells, while others such as CD4^+^ central memory T-cells, late-differentiated CD4^+^ and CD8^+^ TemRA and effector memory T-cells, and CD56^Dim^ NK-cells increased.

### The changes related to CMV and/or EBV seropositivity on CD4^+^ T-cell in blood

First, we focused on differences in CD4^+^ T-cell numbers in CMV and/or EBV seropositive individuals. The reported T-cell and immune cell numbers below were all quantified from blood samples. When only considering CMV serostatus, it appeared that CMV^+^ individuals of all ages had more late-differentiated CD4^+^ TemRA and effector memory cells than non-infected controls. Additionally, CMV^+^ middle-aged adults had more HLA-DR^+^ CD38^−^ CD4^+^ T-cells and CMV^+^ senior adults had increased numbers of HLA-DR^+^ CD38^+/−^ CD4^+^ T-cells both compared to non-infected individuals (Supplementary Fig. S1).

**Figure 1:**
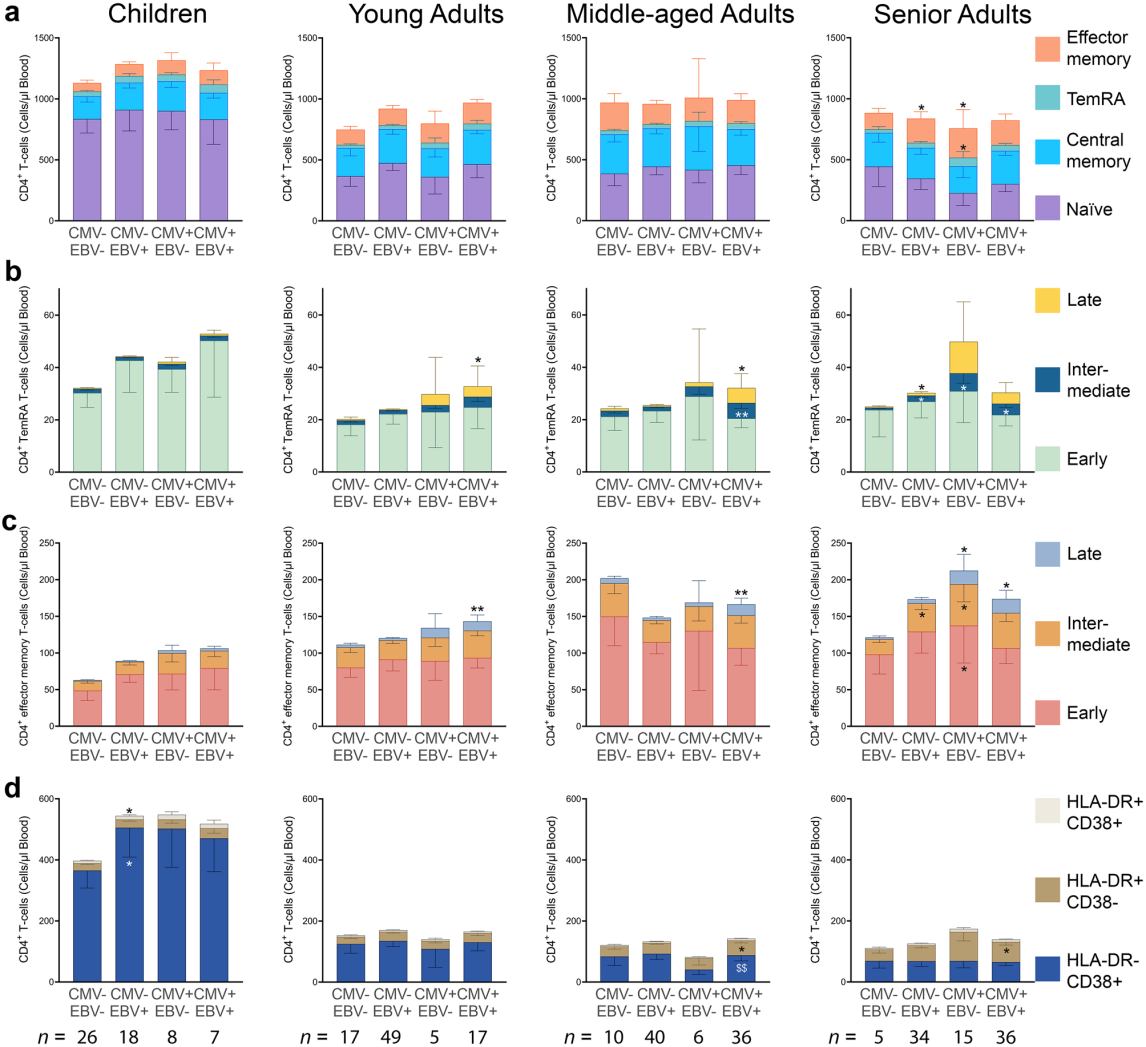
CD4^+^ T-cell numbers in blood of CMV^−^ EBV^−^, CMV^−^ EBV^+^, CMV^+^ EBV^−^, or CMV^+^ EBV^+^ children and young, middle-aged, and senior adults. For these four CMV and EBV seropositivity depending groups, (**a**) numbers of CD4^+^ naïve (CCR7^+^ CD45RA^+^), central memory (CCR7^+^ CD45RA^−^), effector memory (CCR7^−^ CD45RA^−^), or TemRA (CCR7^−^ CD45RA^+^) T-cells are shown, together with numbers of early- (CD45RA^+^ CD28^+^ CD27^+^), intermediate- (CD45RA^+^ CD28^+^ CD27^−^), or late-differentiated (CD45RA^+^ CD28^−^ CD27^−^) (**b**) CD4^+^ TemRA T-cells or (**c**) CD4^+^ effector memory T-cells. (**d**) Differences in numbers of HLA-DR^+/−^ and CD38^+/−^ CD4^+^ T-cell numbers between CMV^−^ EBV^−^, CMV^−^ EBV^+^, CMV^+^ EBV^−^, or CMV^+^ EBV^+^ individuals. Cell numbers are per µl blood and data are geomeans with 95% confidence intervals. A two-way ANOVA with a Dunnett’s multiple comparison test was used as statistical test. *p < 0.05 and ** ^or $$^*p* < 0.01. An asterisk was used to indicate significant differences compared to the CMV^−^ EBV^−^, non-infected group and a dollar sign indicates a comparison with the CMV^+^ EBV^−^ group. *n* = 59 for children, *n* = 88 for young adults, *n* = 92 for middle-aged adults, and *n* = 90 for senior adults. Number of CMV^−^ EBV^−^, CMV^−^ EBV^+^, CMV^+^ EBV^−^, or CMV^+^ EBV^+^ individuals per age group are indicated within the figure.

Next, we separated the study participants based on both CMV and EBV seropositivity (Fig. 1). Firstly, CMV^−^ EBV^+^ senior adults had higher quantities of CD4^+^ effector memory T-cells compared to non-infected senior adults (*p* = 0.048; Fig. 1a). These individuals also had elevated numbers of intermediate- and late-differentiated CD4^+^ TemRA T-cells (*p* = 0.024 and* p* = 0.047, respectively; Fig. 1b) and intermediate-differentiated CD4^+^ effector memory T-cells (and *p* = 0.049; Fig. 1c) in comparison to non-infected individuals. CMV^−^ EBV^+^ children, on the other hand, had elevated numbers of HLA-DR^+/−^ CD38^+^ CD4^+^ T-cells compared to non-infected children (*p* = 0.023 and *p* = 0.028, respectively; Fig. 1d). However, numbers of these cell types were also higher in children seropositive for CMV or both CMV and EBV, although this was not significant due to small group sizes.

CMV^+^ EBV^−^ senior adults had significantly more CD4^+^ TemRA and effector memory cells than non-infected controls (*p* = 0.016 and *p* = 0.025, respectively; Fig. 1a). These differences were the result of CMV^+^ EBV^−^ senior adults having more intermediate-differentiated CD4^+^ TemRA T-cells (*p* = 0.035; Fig. 1b) and more early-, intermediate-, and late-differentiated CD4^+^ effector memory T-cells (all *p* < 0.05; Fig. 1c) than non-infected senior adults.

For young and middle-aged adults, only CMV^+^ EBV^+^ individuals had significant changes in CD4^+^ T-cell numbers. CMV^+^ EBV^+^ young and middle-aged adults had more late-differentiated CD4^+^ TemRA (*p* = 0.027 and *p* = 0.031, respectively; Fig. 1b) and late-differentiated CD4^+^ effector memory cell numbers (*p* = 0.009 and *p* = 0.008, respectively; Fig. 1c) than non-infected controls. Furthermore, compared to non-infected controls, CMV^+^ EBV^+^ middle-aged adults had increased numbers of intermediate-differentiated CD4^+^ TemRA T-cells (*p* = 0.009; Fig. 1b) and of HLA-DR^+^ CD38^−^ CD4^+^ T-cells (*p* = 0.036; Fig. 1d). These individuals also had more HLA-DR^−^ CD38^+^ CD4 T-cells than middle-aged adults only seropositive for CMV (*p* = 0.004), but not compared to non-infected controls.

CMV^+^ EBV^+^ senior adults trended to have more CD4^+^ TemRA T-cells (*p* = 0.063; Fig. 1a) and had significantly more intermediate-differentiated CD4^+^ TemRA T-cells (*p* = 0.020; Fig. 1b) and late-differentiated CD4^+^ effector memory T-cells (*p* = 0.033; Fig. 1c) than non-infected controls. Moreover, compared to CMV^−^ EBV^−^ individuals, increases in HLA-DR^+^ CD38^−^ CD4^+^ T-cell numbers were also observed for CMV^+^ EBV^+^ senior adults (*p* = 0.042; Fig. 1d).

Overall, mostly CMV^+^ EBV^+^ young and middle-aged adults had different quantities of CD4^+^ T-cells compared to non-infected controls, whereas for senior adults differences in numbers of at least one of the analyzed CD4^+^ T-cell types were observed for all infected individuals. As children have more lymphocytes than the older age groups, also CD4^+^ T-cell percentages were analyzed, but this did not reveal any differences between non-infected children and CMV and/or EBV infected children (Supplementary Fig. S2).

### The changes related to CMV and/or EBV seropositivity on CD8^+^ T-cells in blood

When assessing CD8^+^ T-cell subsets, CMV^+^ individuals had higher quantities of CD8^+^ effector memory T-cells and late-differentiated CD8^+^ effector memory and TemRA T-cells than CMV^−^persons (Supplementary Fig. S3). CMV^+^ middle-aged and senior adults also had more CD8^+^ TemRA T-cells and HLA-DR^+^ CD38^+/−^ CD8^+^ T-cells than non-infected controls (Supplementary Fig. S3).

**Figure 2:**
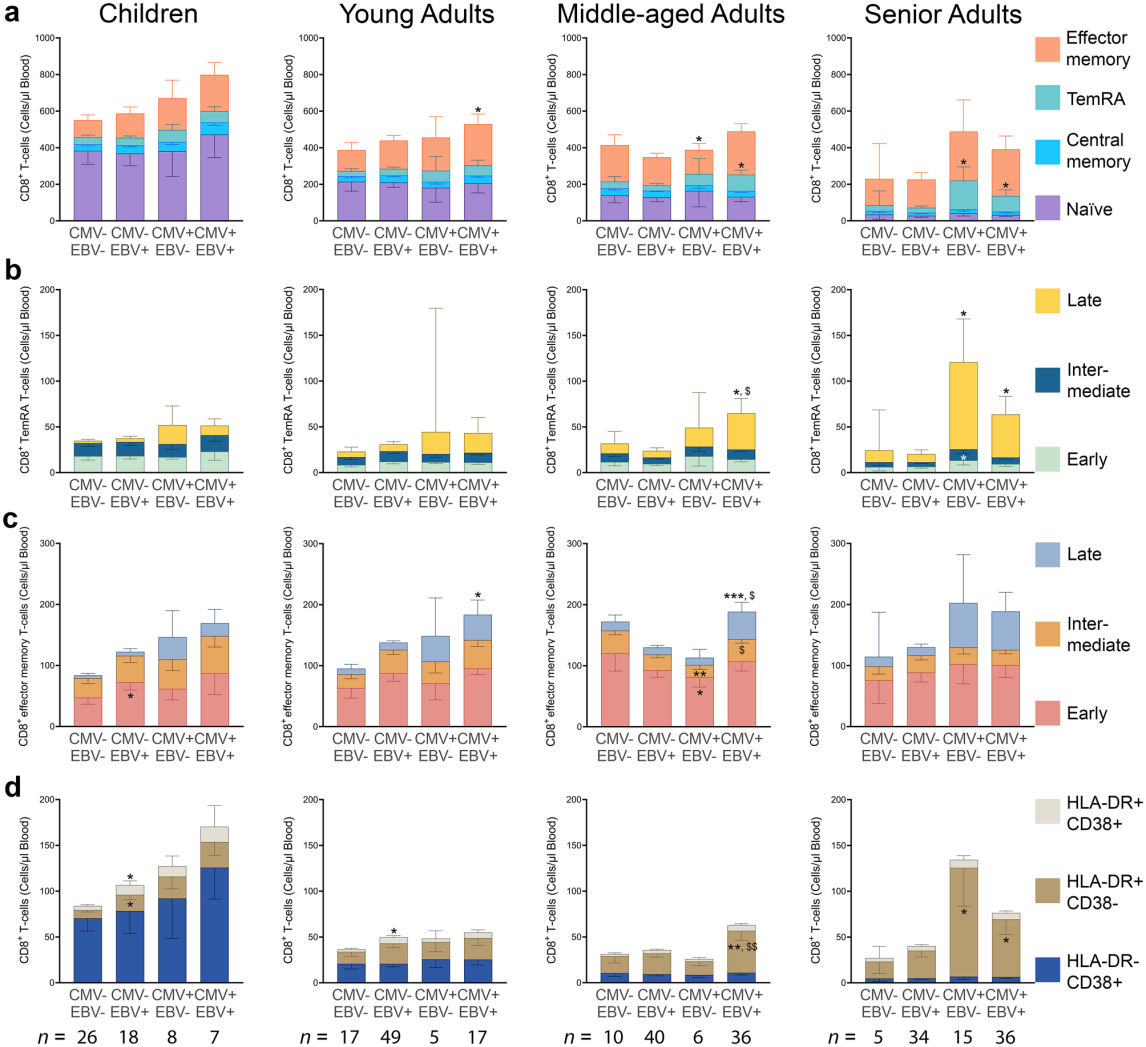
CD8^+^ T-cell numbers in blood of CMV^−^ EBV^−^, CMV^−^ EBV^+^, CMV^+^ EBV^−^, or CMV^+^ EBV^+^ children and young, middle-aged, and senior adults. (**a**) Numbers of CD8^+^ naïve (CCR7^+^ CD45RA^+^), central memory (CCR7^+^ CD45RA^−^), effector memory (CCR7^−^ CD45RA^−^), or TemRA (CCR7^−^ CD45RA^+^) T-cells in CMV^−^ EBV^−^, CMV^−^ EBV^+^, CMV^+^ EBV^−^, or CMV^+^ EBV^+^ individuals. For these four CMV and EBV seropositivity depending groups, numbers of early- (CD45RA^+^ CD28^+^ CD27^+^), intermediate- (CD45RA^+^ CD28^−^ CD27^+^), or late-differentiated (CD45RA^+^ CD28^−^ CD27^−^) (**b**) CD8^+^ TemRA T-cells or (**c**) CD8^+^ effector memory T-cells and (**d**) HLA-DR^+/−^ and CD38^+/−^ CD8^+^ T-cell numbers were determined. Cell numbers are per µl blood and data are geomeans with 95% confidence intervals. * ^or $^*p* < 0.05, ** ^or $$^*p* < 0.01 and ^***^p < 0.001. A two-way ANOVA with a Dunnett’s multiple comparison test was used as statistical test. An asterisk was used to indicate significant differences compared to the CMV^−^ EBV-, non-infected group and a dollar sign indicates a comparison with the CMV^+^ EBV^−^ group. *n* = 59 for children, *n* = 88 for young adults, *n* = 92 for middle-aged adults, and *n* = 90 for senior adults. Number of CMV^−^ EBV^−^, CMV^−^ EBV^+^, CMV^+^ EBV^−^, or CMV^+^ EBV^+^ individuals per age group are indicated within the figure.

Then, the study participants were grouped based on both CMV and EBV serostatus (Fig. 2). CMV^−^ EBV^+^ children had significantly higher numbers of early-differentiated CD8^+^ effector memory T-cells (*p* = 0.034; Fig. 2c) and HLA-DR^+^ CD38^+/−^ CD8^+^ T-cells (*p* = 0.021 and *p* = 0.020, respectively; Fig. 2d) than non-infected children. The increase, although not significant, of the latter cell types was also observed for CMV and/or EBV infected children (Fig. 2d). Additionally, compared to CMV^−^ EBV^−^ young adults, CMV^−^ EBV^+^ young adults had significantly higher numbers of HLA-DR^+^ CD38^+^ CD8^+^ T-cells (*p* = 0.031; Fig. 2d).

CMV^+^ EBV^−^ children and young adults had a significant higher percentage of late-differentiated CD8^+^ TemRA T-cells (*p* < 0.0001 and *p* = 0.003, respectively) and late-differentiated CD8^+^ effector memory T-cells (*p* < 0.0001 and *p* = 0.004, respectively) than non-infected age-matched controls (Supplementary Fig. S4b,c). CMV^+^ EBV^−^ middle-aged adults had significantly lower numbers of CD8^+^ effector memory T-cells compared to non-infected controls (*p* = 0.030; Fig. 2a), which was due to a decrease in early- and intermediate-differentiated CD8^+^ effector memory T-cell numbers (*p* = 0.049 and *p* = 0.004, respectively; Fig. 2c). In contrast, CMV^+^ EBV^−^ senior adults had increased numbers of CD8^+^ TemRA T-cells (*p* = 0.019; Fig. 2a), with more intermediate-and late-differentiated CD8^+^ TemRA T-cells (*p* = 0.024 and *p* = 0.049, respectively; Fig. 2b) compared to non-infected senior adults. CMV^+^ EBV^−^ senior adults also had higher quantities of HLA-DR^+^ CD38^−^ CD8^+^ T-cells than non-infected controls (*p* = 0.022; Fig. 2d).

Most differences were observed for the co-infected individuals. Compared to CMV^−^ EBV^−^ individuals, CMV^+^ EBV^+^ young adults had significantly higher numbers of CD8^+^ effector memory T-cells, whereas CMV^+^ EBV^+^ middle-aged and senior adults had significantly more CD8^+^ TemRA T-cells (*p* = 0.032, *p* = 0.036, and *p* = 0.032, respectively; Fig. 2a). CMV^+^ EBV^+^ young adults also had a higher percentage of late-differentiated CD8^+^ TemRA T-cells (*p* = 0.003; Supplementary Fig. S4b) and more late-differentiated CD8^+^ effector memory T-cells (*p* = 0.037; Fig. 2c) than non-infected controls. Interestingly, CMV^+^ EBV^+^ middle-aged adults had higher numbers of late-differentiated CD8^+^ TemRA T-cells compared to both non-infected and CMV mono-infected controls (*p* = 0.028 and *p* = 0.025, respectively; Fig. 2b). This was also observed for late-differentiated CD8^+^ effector memory T-cell numbers; CMV^+^ EBV^+^ middle-aged adults had significantly more late-differentiated CD8^+^ effector memory T-cells compared to non-infected and CMV^+^ EBV^−^ middle-aged adults (*p* = 0.001 and *p* = 0.012, respectively; Fig. 2c). More HLA-DR^+^ CD38^−^ CD8^+^ T-cells were detected for CMV^+^ EBV^+^ middle-aged compared to non-infected controls and CMV^+^ EBV^−^ middle-aged adults as well (*p* = 0.001 and *p* = 0.009, respectively; Fig. 2d). Lastly, higher numbers of late-differentiated CD8^+^ TemRA T-cells and HLA-DR^+^ CD38^−^ CD8^+^ T-cells were observed for CMV^+^ EBV^+^ senior adults compared to non-infected controls (*p* = 0.047 and *p* = 0.049, respectively; Fig. 2d).

To summarize, differences in CD8^+^ T-cells were detected for CMV^+^ EBV^−^ and CMV^−^ EBV^+^ children and young adults, CMV^+^ EBV^+^ young adults, and for CMV^+^ EBV^−/+^ middle-aged and senior adults. Again, mostly CMV^+^ EBV^+^, and not CMV^+^ EBV^−^, young and middle-aged adults had different CD8^+^ T-cell quantities than non-infected individuals, and for CMV^+^ EBV^+^ middle-aged adults this even significantly differed from CMV^+^ EBV^−^ middle-aged adults.

### The changes related to CMV and/or EBV seropositivity on other immune cells in blood

Furthermore, we also assessed CD4/CD8 ratio’s, granulocyte, NKT, monocyte, Tfh cell, NK-cell, and B-cell numbers for non-infected, CMV-seropositive, and/or EBV-seropositive individuals (Supplementary Figs. S5 and S6). CMV-seropositive senior adults had a lower CD4/CD8 ratio and CMV^+^ middle-aged and senior adults higher NKT cell numbers than CMV^−^ individuals (*p* < 0.0001, *p* = 0.022, and *p* < 0.0001, respectively; Supplementary Fig. S5a). However, when taking into account both CMV and EBV seropositivity, only CMV^+^ EBV^+^ senior adults had significantly more NKT cells compared to non-infected controls (*p* = 0.016; Supplementary Fig. S5b). Moreover, CMV^+^ EBV^+^ children had higher quantities of naïve and CD38^dim^ B-cells than non-infected children (*p* = 0.017 and *p* = 0.016, respectively; Supplementary Fig. S6c).

### Comparability of immune cell composition of CMV and/or EBV seropositive individuals

**Figure 3:**
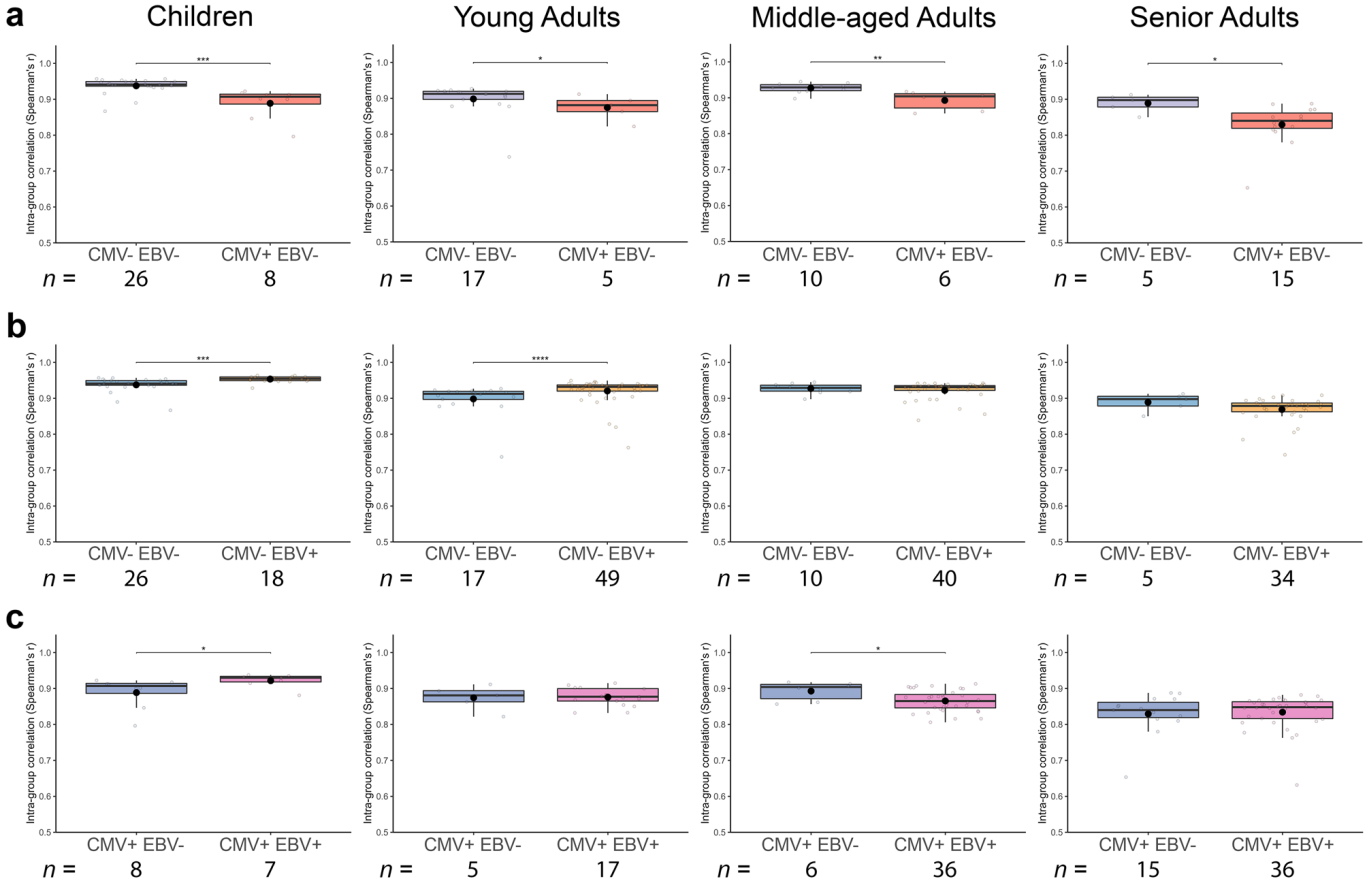
The immune variance between study subjects of the same age category was assessed with an intra-group Spearman correlation analysis. The intra-group correlation coefficients of (**a**) CMV^−^ EBV^−^ and CMV^+^ EBV^−^, (**b**) CMV^−^ EBV^−^ and CMV^−^ EBV^+^, or (**c**) CMV^+^ EBV^−^ and CMV^+^ EBV^+^ individuals are plotted. *p < 0.05, **p < 0.01, ***p < 0.001, and ****p < 0.0001. The number of study participants per group are mentioned within the graph.

Intra-group correlations were performed to assess whether non-infected individuals compared to CMV or EBV mono-infected individuals or the CMV^+^ EBV^−^ group compared to the CMV^+^ EBV^+^ group differed in their immune cell composition. CMV^+^ EBV^−^ individuals of all four age groups had more variance in immune cell composition than CMV^−^ EBV^−^ individuals, resulting in a lower intra-group correlation coefficient (*p* < 0.0001, *p* = 0.038, *p* = 0.007, and *p* = 0.010; Fig. 3a). Interestingly, CMV^−^ EBV^+^ children and young adults had a more equal immune cell composition than non-infected age-matched controls (*p* = 0.0001 and *p* < 0.0001; Fig. 3b). Potentially, this might be due to the expansion of similar cell types because of the EBV infection. A similar result was observed for CMV and EBV co-infected children; these children also had a more equal immune cell composition than CMV^+^ EBV^−^ children (*p* = 0.028; Fig. 3c). In contrast, CMV^+^ EBV^+^ middle-aged adults differed even more in immune cell composition than individuals only seropositive for CMV and had a significantly lower correlation coefficient than CMV^+^ EBV^−^ controls (*p* = 0.048; Fig. 3c).

### Immune cell numbers vs. age, sex, CMV-, or EBV-antibody levels

**Figure 4:**
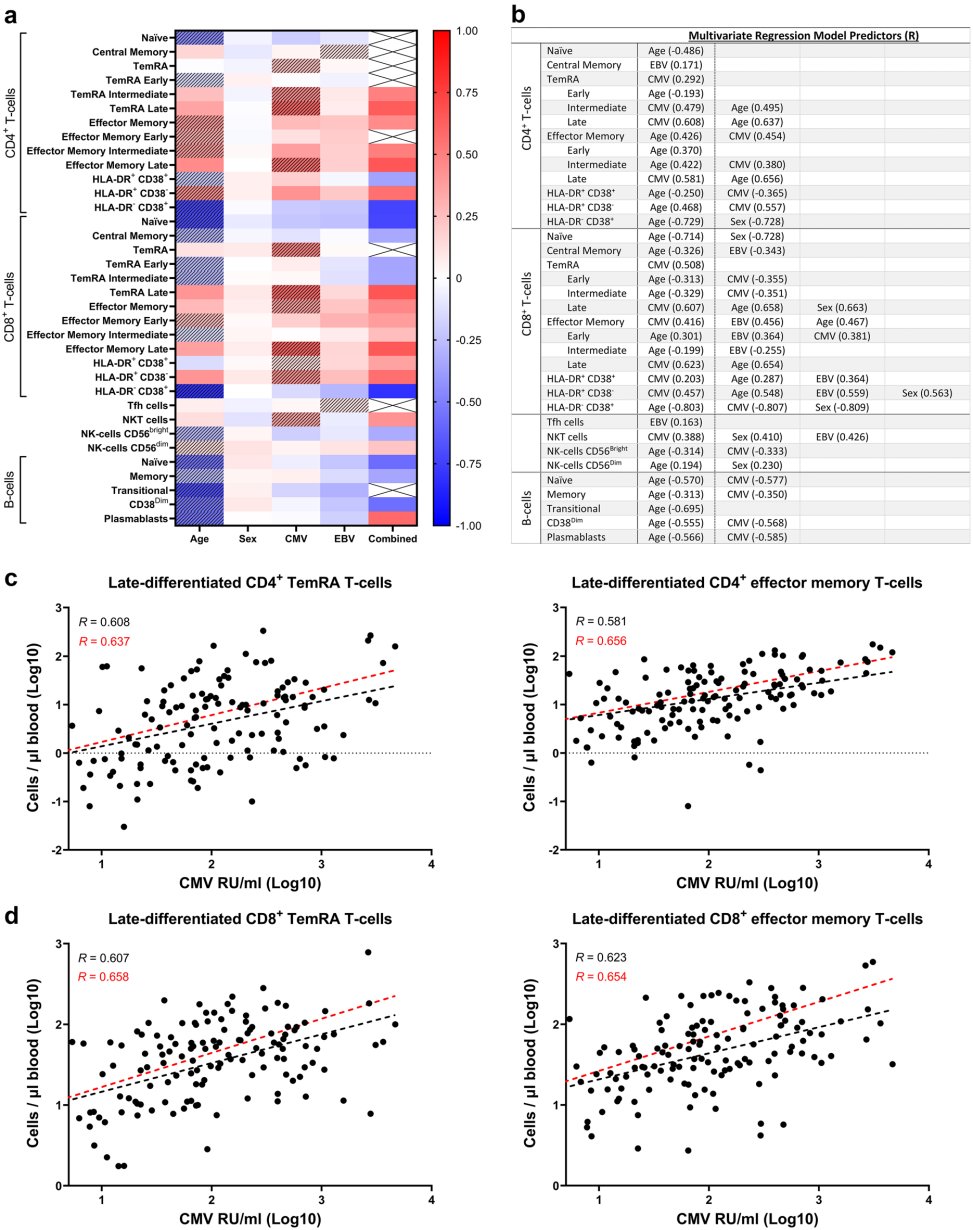
Multivariate regression analysis with absolute immune cell numbers using age, sex, CMV- and EBV-antibody levels as potential predicting factors. (**a**) A heatmap depicting correlation coefficients from the multivariate regression analysis for T-cells, NK-cells, and B-cells per independent predictor being age, sex, CMV- and EBV-antibody levels, or a combination of these factors. Stripes are used to indicate which factor correlated the strongest with quantities of an immune cell and correlation coefficients ranged from 1 to − 1. (**b**) A table summarizing the factor(s) that significantly correlated with the analyzed immune cells. *n* = 336. Correlation coefficients and combined correlation coefficients are given in parentheses. Scatter plots with log transformed anti-CMV antibody levels (RU/ml) and (**c**) late-differentiated CD4^+^ TemRA T-cells or CD4^+^ effector memory T-cells (CCR7^−^ CD45RA^+/−^ CD28^−^ CD27^−^, respectively) or (**d**) late-differentiated CD8^+^ TemRA T-cells or CD8^+^ effector memory T-cells (CCR7^−^ CD45RA^+/−^ CD28^−^ CD27^−^, respectively). *n* = 335, but only data for CMV-seropositive individuals is plotted. Correlation coefficients and linear regression lines with only CMV-antibody levels as predicting factor are shown in black and those with CMV-antibody levels and age as predicting factors are shown in red.

To determine whether CMV- and/or EBV-antibody levels correlated with T-cell, B-cell, NK-cell, and NKT cell numbers regardless of the factors age and sex, a multivariate regression analysis with the predictors age, sex, CMV-, and EBV-antibody levels was performed. Results of this analysis are summarized in a heatmap depicting correlation coefficients per factor or combined correlation coefficients from multiple factors (Fig. 4a) and are enlisted in a table (Fig. 4b). The included immune cell numbers mostly correlated with age or anti-CMV antibody levels, or a combination of these two factors. As main predictor but in combination with other factors, CMV-antibody levels positively correlated with numbers of intermediate-differentiated CD4^+^ TemRA T-cells, CD8^+^ effector memory T-cells, HLA-DR^+^ CD38^+/−^ CD8^+^ T-cells, and NKT cells. Furthermore, anti-CMV antibody levels in combination with age showed a strong and positive correlation with late-differentiated CD4^+^ TemRA and effector memory T-cell numbers (*R* = 0.637 and *R* = 0.656, respectively; Fig. 4c) and late-differentiated CD8^+^ TemRA and effector memory T-cell numbers (*R* = 0.658 and *R* = 0.654, respectively; Fig. 4d).

EBV-antibody levels as an added factor strengthened the positive correlations of CD8^+^ effector memory T-cell, HLA-DR^+^ CD38^+/−^ CD8^+^ T-cell, and NKT cell numbers with CMV-antibody levels. Some immune cells correlated solely with the factors CMV- or EBV- antibody levels. These were CD4^+^ central memory T-cells and Tfh cells, which had a positive, weak correlation with EBV-antibody levels (R = 0.171 and R = 0.163, respectively). CD4^+^ and CD8^+^ TemRA T-cell numbers had a positive correlation with CMV-antibody levels (*R* = 0.292 and *R* = 0.508, respectively).

### Age- and CMV-associated immune phenotypes

**Figure 5:**
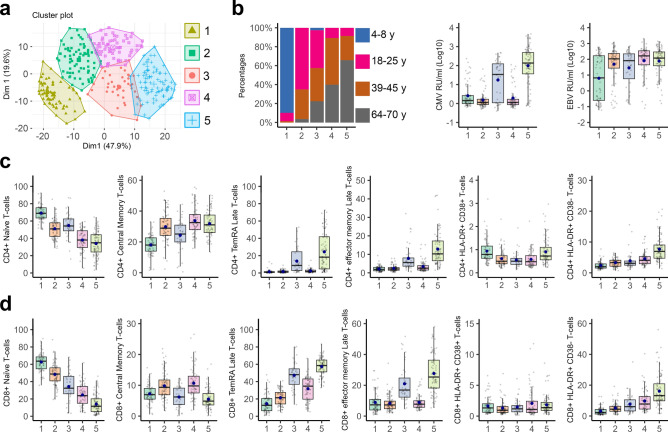
Cluster analysis with percentages of 44 immune cell subpopulations quantified from blood samples of all study participants. (**a**) A principal component analysis plot showing the five clusters the study participants were grouped in. (**b**) Information on age, CMV- and EBV-antibody levels per cluster. Percentages of (**c**) CD4^+^ or (**d**) CD8^+^ naïve (CCR7^+^ CD45RA^+^), central memory (CCR7^+^ CD45RA^−^), late-differentiated TemRA (CD45RA^+^ CD28^−^ CD27^−^), late-differentiated effector memory (CD45RA^−^ CD28^−^ CD27^−^), HLA-DR^+^ and CD38^+^, and HLA-DR^+^ and CD38^−^ T-cells per cluster. *n* = 68 for cluster-1, *n* = 80 for cluster-2, *n* = 40 for cluster-3, *n* = 65 for cluster-4, and *n* = 82 for cluster-5.

Lastly, the study participants were grouped based on their immune cell composition in blood (percentages of 44 immune cell subsets) by performing a gap statistics analysis to test whether the individuals may group differently than grouping based on their chronological age. This analysis showed that the study participants can be subdivided in five clusters (Fig. 5a) and that the clustering of donors mostly depended on age and CMV seropositivity, with some minor differences in sex and EBV seropositivity between the clusters (Fig. 5b & Supplementary Fig. S7a & Table 2). More specifically, children were grouped in cluster-1 regardless of their CMV-serostatus and this cluster had the least EBV-seropositive individuals (compared to cluster-2, -4, and -5; *p* = 0.002, *p* = 0.001, and *p* < 0.001, respectively). Young and middle-aged adults that were CMV-seronegative were present in cluster-2 and cluster-3 contained for the most part young and middle-aged adults that were CMV-seropositive. Cluster-4 consisted mainly of middle-aged and senior adults that were CMV-seronegative. In contrast, cluster-5 had the highest percentage of CMV- and EBV-seropositive individuals (compared to cluster-1, -2, and -4; all *p* < 0.001) and the lowest percentage of female donors (compared to cluster-2 and -4; *p* = 0.024 and *p* = 0.035, respectively), whom were mostly senior adults, but some of them were young and middle-aged adults.

**Table 2:** Demographics of all study participants within the five clusters identified with gap statistics based on the immune cell percentages.

	Cluster-1	Cluster-2	Cluster-3	Cluster-4	Cluster-5
*Characteristics all participants*					
Number (%)	68 (20.3%)	80 (23.9%)	40 (11.9%)	65 (19.4%)	82 (24.5%)
Age (mean ± SD)	8 ± 5.8	29.8 ± 11.9	38.9 ± 18.5	50.6 ± 15	56.4 ± 15.4
Sex (% women)	34 (50%)	55 (68.8%)	28 (70%)	45 (69.2%)	37 (45.1%)*^2,4^
Log_10_ CMV—RU/ml (mean ± SD)	0.41 ± 0.64	0.17 ± 0.41	1.34 ± 0.98**^1,^****^2,4^	0.29 ± 0.59	2.01 ± 1.02****^1,2,4^
BMI (mean ± SD)	16 ± 3.8****^2,3,4,5^	23.4 ± 4.1	22.8 ± 5.2	26.9 ± 5.5**^1,^*^3^	25.9 ± 6.8**^2,^*^3^
Unhealthy BMI (%)	4 (5.9%)**^4,^***^5^	19 (24.6%)**^4,^***^5^	10 (28.6%)**^5^	34 (57.6%)**^1,2^	51 (66.2%)***^1,2,^**^3^
Smoking (% yes)	1 (1.5%)	9 (11.3%)	5 (12.5%)	11 (16.9%)	9 (11%)
Score risk diseases (%)†	0 (0%)‡	1 (0.3%)***^4,5^	4 (2.5%)*^5^	15 (5.8%)***^2^	27 (8.2%)***^2,^*^3^
Score acute clinical symptoms (%)†	0 (0%)	72 (34.8%)	14 (16.7%)	49 (30.2%)	52 (29.4%)
*Seroprevalence all participants (number (%))*					
CMV^+^	15 (22.1%)***^3,5^	8 (10%)***^3,5^	28 (70%)****^1,2,4^	11 (16.9%)***^3,5^	70 (85.4%)***^1,2,4^
EBV^+^	31 (45.6%)**^2,4,5^	61 (76.3%)**^1^	26 (65%)	52 (80%)**^1^	66 (80.5%)**^1^
CMV^−^ EBV^−^	27 (39.7%)**^3,4,^***^5^	17 (21.3%)**^5^	4 (10%)	8 (12.3%)	2 (2.4%)
CMV^+^ EBV^+^	7 (10.3%)	7 (8.8%)	19 (47.5%)***^1,2,4^	7 (10.8%)	56 (68.3%)***^1,2,4^
VZV^+^	58 (85.3%)	79 (98.8%)	40 (100%)	65 (100%)	82 (100%)
*Characteristics CMV*^*+*^* EBV*^*+*^* young and middle-aged adults*					
Number (%)	0 (0%)	7 (13.2%)	16 (30.2%)	5 (9.4%)	25 (47.2%)
Age (mean ± SD)	– ± –	25.9 ± 9.5	33.4 ± 10.2	43.4 ± 1.4	36 ± 9.2
Sex (% women)	0 (0%)	4 (57.1%)	14 (87.5%)*^5^	3 (60%)	12 (48%)
Log_10_ CMV—RU/ml (mean ± SD)	– ± –	1.29 ± 0.31	1.99 ± 0.41	1.67 ± 0.46	2.33 ± 0.64****^2,4^
BMI (mean ± SD)	– ± –	23.9 ± 2.9	23.3 ± 3.1	24.4 ± 2.8	25.4 ± 4.7
Unhealthy BMI (%)	0 (0%)	2 (33.3%)	3 (21.4%)	2 (40%)	10 (50%)
Smoking (% yes)	0 (0%)	1 (14.3%)	2 (12.5%)	2 (40%)	3 (12%)
Score risk diseases (%)†	0 (0%)	0 (0%)	1 (2.1%)	0 (0%)	0 (0%)
Score acute clinical symptoms (%)†	0 (0%)	7 (46.7%)	9 (27.3%)	6 (40%)	24 (44.4%)

Additionally, characteristics of CMV^+^ EBV^+^ young and middle-aged adults present within the five clusters are enlisted. Data in bold was significantly higher and data in bold and underlined was significantly lower. In superscript it is indicated whether the value was significantly different from cluster-1, cluster-2, cluster-3, cluster-4, or cluster-5. *p < 0.05, **p < 0.01, ***p < 0.001, and ****p < 0.0001. ‡Not used within the Chi-square analysis. †Reported numbers are the sum of the weighted scores and the percentages are the percentage from the highest possible score.

Based on the immune cell percentages, individuals in cluster-5 had the most differential immunotype compared to individuals from the other clusters (Supplementary Fig. S7b), with the largest differences being within T-cell subset percentages (Fig. 5c,d). The donors from cluster-5 had a significantly lower percentage of CD8^+^ naïve T-cells (compared to clusters-1, -2, and -4), CD4^+^ naïve T-cells (compared to cluster-1 and -3), and CD8^+^ central memory T-cells (compared to cluster-4). In contrast, individuals from cluster-5 had a significantly higher percentage of late-differentiated CD4^+^ and CD8^+^ TemRA and effector memory T-cells (compared to clusters-1, -2, and -4), and HLA-DR^+^ CD38^−^ CD4^+^ and CD8^+^ T-cells (compared to cluster-1, -2, and -3; Supplementary Fig. S7c).

Between the different clusters, there were also some differences in the number of participants with an unhealthy BMI and the score of risk diseases that includes having diabetes, rheumatoid arthritis, vascular diseases, or cancer and which are potentially associated with CMV seropositivity (Table 2). Cluster-1 had the least individuals with an unhealthy BMI (compared to cluster-4 and -5; *p* = 0.005 and *p* < 0.001, respectively) and the lowest score risk diseases compared to the other clusters. Cluster-5 on the other hand contained significantly more individuals with an unhealthy BMI (compared to cluster-1, -2, and -3; *p* < 0.001, *p* < 0.001, and *p* = 0.003, respectively) and a high score risk diseases (compared to cluster-2 and -3; *p* < 0.001 and *p* = 0.038, respectively).

### Comparison between CMV^+^ EBV^+^ young and middle-aged adults of the five different clusters

Importantly, cluster-5, which was the most differential immunotype, was not only formed by senior adults, but also by some of the young and middle-aged adults. These individuals were mainly seropositive for both CMV and EBV. CMV- and EBV-seropositive young and middle-aged adults were also present in cluster-2, -3, and -4 (Table 2). However, these CMV^+^ EBV^+^ individuals of cluster-2 and cluster-4 had significantly lower CMV-antibody levels than CMV^+^ EBV^+^ young and middle-aged adults in cluster-5 (*p* < 0.0001 and *p* = 0.042, respectively). The CMV^+^ EBV^+^ young and middle-aged adults from cluster-3 had not only a slightly lower anti-CMV antibody levels (*p* = 0.142) but also tended to have a healthier BMI (*p* = 0.092) than age-matched individuals from cluster-5. Also, cluster-3 had significantly more female CMV^+^ EBV^+^ young and middle-aged adults than cluster-5 (*p* = 0.010), although this should be interpreted with caution given that the number of 18–25 y and 39–45 y old participant per cluster was not equal. So, it appeared that for some CMV and EBV co-infected young and middle-aged adults had a T-cell composition that resembles that of senior adults.

## Discussion

**Figure 6:**
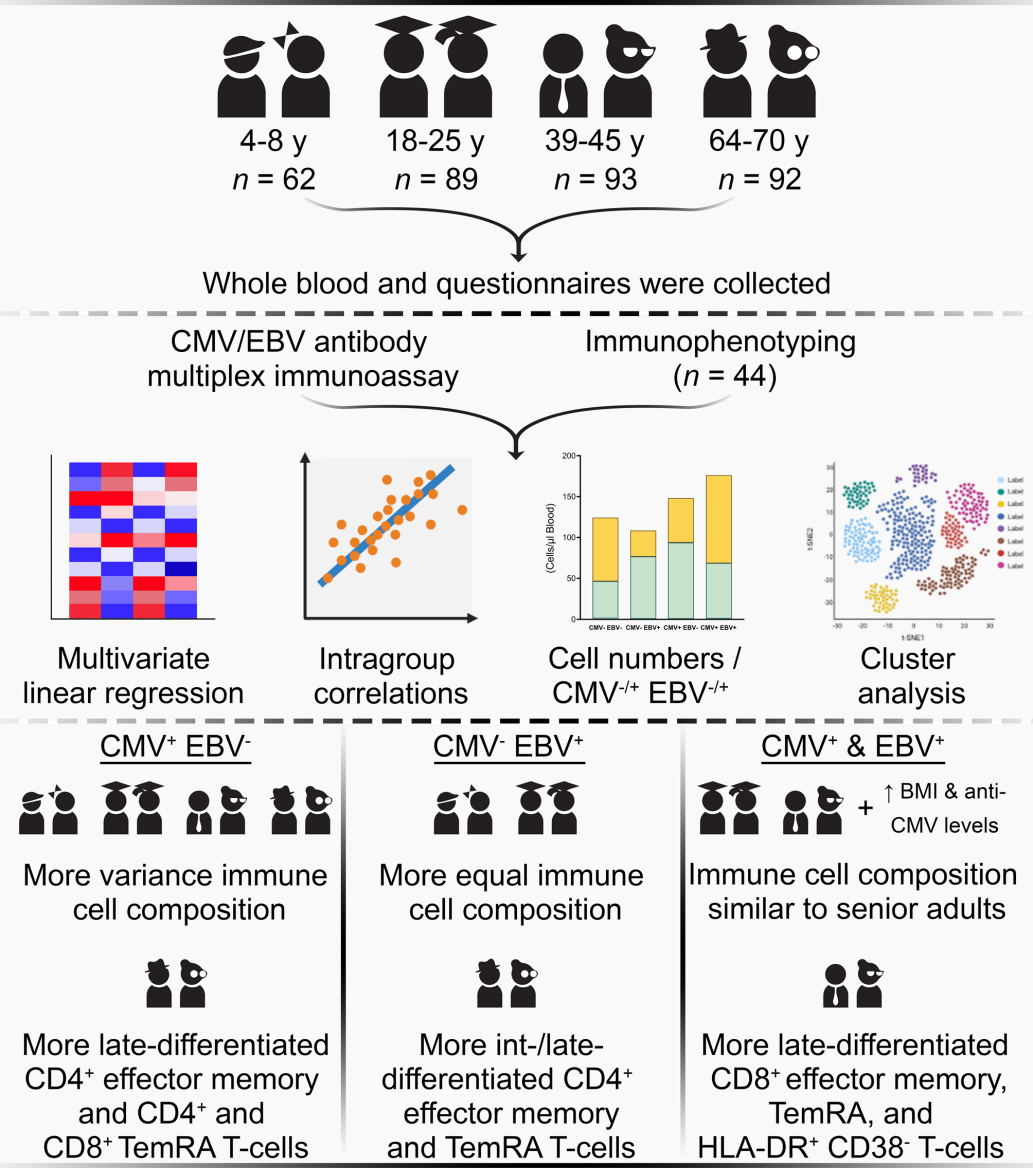
Schematic study design with an overview of the performed analyses and the main findings of this study. This image was partially created with BioRender.com.

In this paper, we investigated the changes in quantities of T-cells and other immune cells in blood of CMV or EBV mono-infected or CMV and EBV co-infected children, young adults, middle-aged adults, and senior adults. We found that CMV^+^ EBV^−^ individuals of all age groups had more variance in their immune cell composition in blood than non-infected individuals, and late-differentiated CD4^+^ and CD8^+^ effector memory and TemRA T-cell numbers correlated strongly with anti-CMV antibody levels in combination with age. We also showed that CMV^−^ EBV^+^ children, young adults, and senior adults had differences in T-cells quantities compared to non-infected age-matched controls, and that CMV and EBV co-infected middle-aged adults had more late-differentiated CD8^+^ effector memory and TemRA T-cells and HLA-DR^+^ CD38^−^ CD8^+^ T-cells in comparison to a CMV mono-infected middle-aged adults. Furthermore, we could cluster the study participants based on their immune cell composition in blood by utilizing a cluster analysis. This clustering of the study participants mostly depended on the factors age and CMV seropositivity, and it allowed us to study immune variance of our study population even in more depth. Also, from this analysis, it became apparent that some CMV and EBV co-infected young and middle-aged adults whom tended to have an unhealthier BMI and higher anti-CMV antibody levels had an immune cell composition similar to senior adults (Fig. 6).

One study previously reported that a CMV and EBV co-infection in 6-year old children can have additive effects on effector memory T-cell expansion compared to a latent CMV infection^21^. There were no differences in T-cell numbers between CMV^+^ EBV^+^ children and CMV mono-infected children in this study, which might be due to having a smaller sample size, but we did observe that CMV^+^ EBV^+^ children had lower intra-group immune variation than CMV^+^ EBV^−^ children. Additionally, this current study did show that CMV^+^ EBV^+^ middle-aged adults had a larger expansion of late-differentiated CD8^+^ effector memory and TemRA T-cells and HLA-DR^+^ CD38^−^ CD8^+^ T-cells than CMV single infected middle-aged adults. Recent reports observed HLA-DR expressing CD8^+^ T-cells, which also expressed the exhaustion markers PD-1 and TIGIT, to have a regulatory phenotype, while these cells also secreted IFN-γ and TNF-α^24,25^. It would be interesting to investigate this in future together with whether T-cells of CMV^+^ EBV^+^, CMV^+^ EBV^−^, or CMV^−^ EBV^+^ individuals differ in function, as these above-mentioned differences were not maintained for CMV^+^ EBV^+^ senior adults.

Besides CMV-seropositive people having more late-differentiated CD4^+^ and CD8^+^ effector memory and TemRA T-cell and NKT cell numbers, which are in line with previous reports^5–7,10,11^, we observed that EBV mono-infected senior adults had higher numbers of intermediate- and late-differentiated TemRA CD4^+^ T-cells and intermediate-differentiated effector memory CD4^+^ T-cells compared to non-infected senior adults. Previous reports also showed that an EBV infection leads to the expansion of more early- and intermediate-differentiated T-cells rather than late-differentiated T-cells^19,20,26^. Although these reports did focus on CD8^+^ T-cells and showed that these EBV-specific T-cells mostly expressed CD45RO^19,20,26^. Interestingly, re-expression of CD45RA was observed during lytic responses; intermediate-differentiated CD8^+^ TemRA cells expanded after exposure to lytic EBV peptides ex vivo^20^.

This current paper showed that CMV^−^ EBV^+^ children and young adults had a more homogeneous immune cell composition than EBV-seronegative children and young adults. Also, compared to non-infected children, the EBV-seropositive children had more HLA-DR^+^ CD38^−/+^ CD4^+^ T-cells, HLA-DR^+^ CD38^−^ CD8^+^ T-cells, and early-differentiated CD8^+^ effector memory T-cells, and both CMV^−^ EBV^+^ children and young adults more HLA-DR^+^ CD38^+^ CD8^+^ T-cells. The elevated prevalence of these cells might explain the decrease in variation among these children’s and young adult’s immune cell composition. As mentioned above, HLA-DR^+^ CD8^+^ T-cells might have regulatory functions and express exhaustion markers. A recent study in mice showed that CD38^+^ CD8^+^ T-cells present during an acute or chronic infections can also expressed the exhaustion marker PD-1 and that CD38 expression, especially during a chronic CMV infection, lowered Granzyme B production and proliferation of the T-cells while supporting survival of these cells^27^. Potentially, the role of these HLA-DR^+^ and/or CD38^+^ T-cells is to suppress immune responses during chronic infection. Additionally, a large cohort study did find that blood samples of EBV-seropositive children had more CD8^+^ effector memory and TemRA T-cells than non-infected children^21^, but we did not observe this potentially since we have a smaller sample size.

Furthermore, when clustering individuals based on their immune cell composition in blood, it was also revealed that some CMV^+^ EBV^+^ young and middle-aged adults had a more altered immune phenotype, similar to CMV^+^ EBV^−/+^ senior adults, with high numbers of CD4^+^ and CD8^+^ effector memory T-cells and T-cells expressing HLA-DR, but low numbers of CD4^+^ and CD8^+^ naïve T-cells than age-matched individuals in other clusters. Recently, there is more and more research performed to find biomarkers to estimate which individuals differ regarding their immune variance compared to individuals of the same age. Some recent publications refer to this as the biological age of a person^28,29^. For example, the inflammatory aging clock (iAGE) based on soluble immune biomarkers such as cytokine levels and immune cell quantities and clinical questionnaires^29^ or the aging-related immune phenotype (ARIP) which specifically looked at CD4^+^ and CD8^+^ T-cell subset quantities^28^. Interestingly, the latter study found that CD4^+^ naïve T-cells numbers or the ratio between CD4^+^ naïve and central memory T-cells correlated the strongest with the biological age of the study participants^28^. Also the individuals from cluster-5, being the CMV^+^ EBV^+^ young and middle-aged adults clustering together with the senior adults, had significantly lower CD4^+^ naïve T-cells than cluster-3, which mostly contained CMV^+^ EBV^−/+^ young and middle-aged adults. Whether having an immune cell composition that is similar to senior adults as a young and middle-aged adult is also associated with being less immunocompetent needs to be further investigated.

These CMV^+^ EBV^+^ young and middle-aged adults that clustered with senior adults tended to have a more unhealthy BMI. A direct link between being overweight or obese and T-cell differentiation has been suggested before^30^. More specifically, excess body mass correlated positively with proportions of late-differentiated and CD57 expressing CD4^+^ and CD8^+^ T-cells and negatively with proportions of naïve T-cells, regardless of the age, CMV- or EBV-serostatus^30^. Furthermore, an unhealthy BMI and more adipose tissue has previously been associated with a systemic “low-grade” inflammation given that adipocytes and innate immune cells secrete more IL-6, TNF-α, and IL-1β^31,32^. Potentially, these higher levels of IL-6 might enable reactivation of CMV as shown to occur in vitro^1,33^, which then indirectly promotes the expansion of more late-differentiated effector memory T-cells.

Since CMV-specific antibody levels might be indicative for more reactivation of the virus^34,35^, it is interesting that we observed slightly higher anti-CMV antibody for CMV^+^ and EBV^+^ young and middle-aged adults that clustered with CMV^+^ and EBV^−/+^ senior adults than CMV^+^ EBV^+^ age-matched individuals from other clusters. Additionally, others have proposed that during a CMV and EBV co-infection, EBV has the possibility the reactivate easier^36^ as there is a decrease in EBV-specific CD8^+^ T-cell numbers^20^ with a less diverse TCR receptor^37^. Potentially during a CMV and EBV co-infection there is reactivation of one or both of the herpesviruses, which increases their viral load in the host. Consequently, this may trigger the expansion of even more effector memory T-cells in an attempt to control the viral reactivation, resulting in differences in T-cell numbers between individuals with a CMV and EBV co-infection or a mono-infection with just CMV.

Whether the reported differences in T-cell quantities between CMV and EBV co-infected or CMV mono-infected young and middle-aged adults has any physiological implications remains a question. A recent publication did shown CMV^+^ individuals to have reduced antigen-specific responsiveness (less IFN-γ secreting CD4^+^ T-cells and less neutralizing antibodies) to de novo immunization with a tick-borne encephalitis vaccine compared to CMV^−^ individuals^4^. Further, the number of influenza-specific CD8^+^ T-cells have been reported to decline with age for CMV^+^ individuals, but not for CMV^−^ individuals^38^. Nevertheless, humoral responses to immunization with an influenza vaccine did not differ between CMV seropositive or negative persons, and CMV^+^ individuals appeared to even be more protected after this vaccine than CMV-seronegative adults^39^. Also in children a CMV infection did not cause lower antibody responses after measles vaccination while an EBV infection did, and children with a CMV and EBV co-infection did have good antibody responses^40^. Similarly, a co-infection with both CMV and EBV also rescued the negative effect of EBV on vaccine-specific IgG titer decay rates^41^. Future research is needed to clarify the implications or benefits of having these herpesvirus infections.

Also the timing and the duration of the primary CMV infection have been previously correlated to the degree of expansion of effector memory and TemRA T-cells^42^. Older adults with a short-term CMV infection had more CD4^+^ effector memory and TemRA T-cells than individuals that had a long-term CMV infection^42^. This may explain why more of the differences in T-cell numbers were observed in the older age groups as children and young adults are more likely to have a short-term CMV infection. Furthermore, CMV seroconversion at an older age (≥ 45 years of age) also resulted in higher CD4^+^ effector memory and TemRA T-cell quantities than CMV seroconversion at a younger age (< 38 years of age)^42^. Potentially, younger individuals need less effector memory and TemRA T-cells to control the virus or may be even better equipped to control viral loads. From studies in mice it is known that the size of the primary viral dose does determine the magnitude of T-cells to expand and their differentiation level^43,44^. In this current study we do not have information on the viral inoculum of the initial infection or on when the primary infections occurred. For the latter, a longitudinal study could potentially give more insight into what causes some CMV and EBV co-infected individuals to have a more alter T-cell composition than others.

Similar to an earlier report^23^, when clustering the study participants based on their immune cell composition in blood, age and CMV seropositivity are important factors of how the clusters are formed. Although CMV-seropositive children had more immune variance than CMV^−^ children, children were not separated into two different clusters with CMV-seropositive children and CMV-seronegative children. An explanation for this might be that the immune cell composition of children is vastly different from the adults that subtle nuances in immune cell numbers for children were not strong enough to form distinct clusters.

To conclude, this study identified differences in quantities of intermediate- or late-differentiated CD4^+^ and CD8^+^ effector memory and TemRA T-cells and T-cells that expressed HLA-DR and/or CD38 in blood samples of CMV- and/or EBV-seropositive individuals. Moreover, we showed that CMV and EBV co-infected middle-aged adults and to some extent in young adults can have a more altered T-cell composition that CMV mono-infected young and middle-aged adults, which appeared to be influenced by the height of the anti-CMV antibody levels and excessive body weight. These results provide more insight into immune cell compositions of persons seropositive for CMV, EBV, or both and help us better understand which individuals and under which circumstances are more at risk of being negatively impacted by a latent infection with these herpesviruses.

## Methods

### Study population and blood collection

This study was part of a large Dutch cross-sectional population study over all ages (Pienter3 study) and for which forty municipalities were sampled within five regions proportional to size^45^. This study was conducted in accordance with the Declaration of Helsinki and was approved by the ‘The Medical Ethics Committee Noord-Holland’ in the Netherlands (METC Number: ISRCTN 20164309 and M015–022). After written informed consent was obtained, two peripheral blood samples were collected into heparin containing VACUETTE® tubes (Greiner Bio-one B.V., Alphen a/d Rijn, the Netherlands) from 338 participants, being 4–8 year, 18–25 year, 39–45 year, or 64–70 year of age, in the period between September 2016 and October 2017. For children, the age range 4–8 year was chosen to not have interference with the National Immunization Program. For the young adults, middle-aged adults, and senior adults, small age ranges were chosen to limit variation within the groups since heterogeneity in both immune responsiveness and numbers of different immune cells increases with age. Two participants were excluded given that the age criteria mentioned above was not met (total *n* = 336). The whole blood samples were pooled; one part was used for subsequent antibody stainings and flow cytometric analysis and the second part was centrifuged at 800 × *g* for 10 min at room temperature (RT), plasma was recovered, and the plasma was stored at − 20 °C until further use.

The study participants filled in questionnaires to obtain information about their height, weight and whether they smoked, had any diseases, or any acute symptoms (fever, cough, or nasal congestion) at the moment of sample collection. BMI was calculated with height and weight information and it was evaluated whether a person had an unhealthy BMI yes or no (higher than 24.9 for all study participants or lower than 18.5 for adults or lower than 13.6 for children). A cumulative risk disease score, also referred to as “Score Risk Diseases”, was calculated for the diseases that may be influenced by CMV seropositivity, which included diabetes^46,47^, rheumatoid arthritis^48^, vascular diseases^42,49^, or cancer^50^. This risk disease score ranged from 0 (no diseases) to 4 (all four risk diseases). Also, a cumulative combined acute symptom score (also referred to as “Score Acute Clinical Symptoms”) was also calculated and ranged from 0 (no acute symptoms) to 3 (all acute symptoms). Both the Score Risk Diseases and Score Acute Clinical Symptoms were weighted scores (i.e., a score of 1 × 1, a score of 2 × 2, a score of 3 × 3, and, if applicable, a score of 4 × 4). A sum of the weighted scores was calculated per age group or cluster and divided by the number of people within the group multiplied by the highest possible score (4 for the Score Risk Disease and 3 for the Score Acute Clinical Symptoms) to generate a percentage from the highest possible score. Both the sum of the weighted scores and the percentage from the highest possible score have been reported.

### Immunophenotyping

Antibody mixes for two different flow cytometry panels were prepared in FACS buffer consisting of PBS (without Ca/Mg; Gibco as part of Thermo Fisher Scientific, Bleiswijk, NL) with 0.5% bovine serum albumin (BSA; Sigma-Aldrich, Zwijndrecht, NL) and 2 mM ethylenediaminetetraacetic acid (EDTA; Thermo Fisher Scientific) in 1)BD Trucount tubes (BD Biosciences) or 2) 5 ml polystyrene Falcon tubes (Thermo Fisher Scientific) with 10 µl Brilliant stain buffer (BD Biosciences, Franklin Lakes, NJ, USA) per antibody mix (Supplementary Table S2). Heparin blood (100 µl) was added per antibody mix, samples were incubated for 30 min at RT in the dark, and afterwards, red blood cells were lysed with a 10 × diluted BD FACS lysis buffer (BD Biosciences) for 15 min at RT in the dark. Lastly, samples that received antibody mixes of flow panel 2 were centrifuged at 300 × *g* for 8 min at RT, 600 µl supernatant was removed, the cell pellet was resuspended in the remaining 200 µl, and the cells from both panels were acquired with a LSRII Fortessa X20 flow cytometer (BD Biosciences). Absolute cell numbers for the samples in the non-Trucount tubes were calculated based on the percentage of CD3^+^ cells in both tubes and the absolute number of CD3^+^ cells in the Trucount tube.

A previously published gating strategy was used^51^; T-cells subsets were gated based on CCR7 and CD45RA expression^52^ and gating of early-, intermediate-, and late-differentiated effector memory and TemRA T-cell was done with the markers CD28 and CD27^53^. See Supplementary Fig. S8a and b (panel 1 and panel 2, respectively) for the full gating strategy used for this study. In short, within the CD3^+^ population, the distinguishment between naïve (CCR7^+^ CD45RA^+^), central memory (CCR7^+^ CD45RA^−^), TemRA (CCR7^−^ CD45RA^+^), and effector memory (CCR7^−^ CD45RA^−^) T-cells that were CD4 or CD8 positive was made. Furthermore, the differentiation status, being either early (CD28^+^ CD27^+^), intermediate (CD28^+^ CD27^−^ for CD4^+^ and CD28^−^ CD27^+^ for CD8^+^), or late (CD28^−^ CD27^−^), were determined for TemRA and effector memory T-cells and the expression of the activation markers HLA-DR and CD38 by T-cells was measured. Within the CD19^+^ cell population, the B-cell subsets being naïve (CD27^−^), memory (CD27^+^), transitional (CD38^Bright^ CD27^−^), CD38^Dim^, and plasmablasts (CD38^Bright^ CD27^+^) were gated. NKT cells were CD3^+^ and CD56^+^, whereas T follicular helper cells (Tfh cells) were CD4^+^ CD45RA^−^ and CXCR5^+^. From the CD3^−^CD19^−^ cell potion, NK-cells were gated and were either CD16^Dim/−^ and CD56^Bright^ or CD16^Bright^ and CD56^Dim^. Supplementary Table S3 summarizes all immune cells with their corresponding surface markers quantified with flow cytometry.

### Serology

Anti-CMV and anti-EBV antibody levels were measured in plasma samples of the study participants with an in-house developed multiplex immuno-assay (MIA)^54^. A serum sample (collected for the complete Pienter3 study) was used instead for eight subjects. Individuals were CMV-seropositive with a level of more than 5 relative units (RU)/ml^55^, whereas the threshold for EBV seropositivity was 22 RU/ml and individuals were considered EBV-seronegative with a level ≤ 16 RU/ml. Subjects (*n* = 3) with anti-EBV-antibody levels between 16 – 22 RU/ml were excluded from further analysis for which EBV-serostatus was of importance. For three individuals, no anti-EBV antibody levels were available and, thus, were excluded from analysis where EBV-serostatus was of importance. For one participant, there was no plasma or serum to conduct the MIA with and this participant was not included in analyses for which a CMV- or EBV-serostatus was required.

### Intra-group Spearman correlations and cluster analysis

Intra-group Spearman correlations and a cluster analysis was performed as recently described^23^. In short, a Spearman correlation matrix was generated to correlate the donors based on percentages of 44 immune cell subsets (data were scaled). The Spearman correlation coefficients per individuals were used to assess immune variance between the following groups: (1) CMV^−^ EBV^−^ versus CMV^+^ EBV^−^, (2) CMV^−^ EBV^−^ versus CMV^−^ EBV^+^, or (3) CMV^+^ EBV^−^ versus CMV^+^ EBV^+^. Additionally, with the correlation matrix, gap statistics was performed to determine the optimal number of clusters needed to group the donors^56^ and, afterwards, the data was clustered with k-means clustering^57^.

### Data analysis

Data from flow cytometric analysis were evaluated with FlowJo 10.8.0 (FlowJo company, Ashland, OR, USA). Data visualization and statistical tests were done in GraphPad Prism version 9.3.1 (GraphPad Software Inc, San Diego, CA, USA), unless stated otherwise. Normality of the data was checked with a Shapiro–Wilk test. Differences in immune cell quantities between the four age groups were analyzed with a non-parametric Kruskall-Wallis test combined with a Dunn’s multiple comparison test corrected for multiple comparisons. When CMV or EBV seropositivity was taken into account, a two-way ANOVA with a Dunnett’s multiple comparison test that was corrected for multiple comparisons was used. Statistics with T-cell percentages was performed with a two-way ANOVA and Holm-Bonferroni post-hoc test in R 4.2.0 and R studio 2022.02.2.

A multivariate linear regression was performed to determine whether the factors age, sex, log transformed anti-CMV or anti-EBV antibody levels potentially correlate with flow cytometric data, being absolute cell numbers of different immune cells, by adding these factors in a stepwise manner to the regression equation. The software IBM SPSS Statistics 28 (IBM, Armonk, NY, USA) was used for this.

The clustering analyses of donors based on immune cell percentages and visualization of this data was done in R 4.2.0 and R studio 2022.02.2. Different R packages were used for this; stats (v4.2.0) to generate a correlation matrix (method = Spearman), scales (v1.2.1) for scaling the correlation data, cluster (v2.1.4) for gap statistics (k.max = 10, nboot = 500, nstart = 25), factoextra (v1.0.7) for visualization of initial gap statistics results (method = Tibs2001SEmax), stats for k-means clustering (nstart = 100, algorithm = Hartigan-Wong), ggplot2 (v3.3.6) for generating the graphs containing information on the clusters. A non-parametric Kruskall–Wallis test with a Dunn’s multiple comparison test (all participants) or a parametric one-way ANOVA with a Sidak’s multiple comparison (CMV^+^ EBV^+^ 18–25 year and 39–45 year old individuals) test, both corrected for multiple comparisons, was used to assess differences in BMI and log transformed anti-CMV antibody level data between clusters. Differences in categorical data between clusters were evaluated with a Pearson chi-square test combined with a post-hoc test and Bonferroni correction with IBM SPSS Statistics software. Volcano plots were generated in R studio with the ggplot2 package and contained information on *p* values from a one-way ANOVA with a TukeyHSD post-hoc test compared to cluster 4 and immune cell percentage fold changes also compared to cluster 4. Statistical analyses were performed with the R packages stats or Rstatix (v 0.7.0) and the R package ggpubr (v 0.4.0) was used to visualize *p* values for the intra-group correlation plots. Analysis with *p* values of < 0.05 were considered statistically significant.

## Supplementary Information

Supplementary Information.

## Data Availability

The datasets generated during and/or analyzed during the current study are available from the corresponding author on reasonable request.

## References

[1] Nikolich-Žugich J (2020). Advances in cytomegalovirus (CMV) biology and its relationship to health, diseases, and aging. Geroscience.

[2] Poole E, Sinclair J (2015). Sleepless latency of human cytomegalovirus. Med. Microbiol. Immunol..

[3] Myerson D, Hackman RC, Nelson JA, Ward DC, McDougall JK (1984). Widespread presence of histologically occult cytomegalovirus. Hum. Pathol..

[4] Nicoli F (2022). Primary immune responses are negatively impacted by persistent herpesvirus infections in older people: Results from an observational study on healthy subjects and a vaccination trial on subjects aged more than 70 years old. EBioMedicine.

[5] Derhovanessian E (2011). Infection with cytomegalovirus but not herpes simplex virus induces the accumulation of late-differentiated CD4^+^ and CD8^+^ T-cells in humans. J. Gen. Virol..

[6] Derhovanessian E (2013). Lower proportion of naïve peripheral CD8^+^ T cells and an unopposed pro-inflammatory response to human Cytomegalovirus proteins in vitro are associated with longer survival in very elderly people. Age.

[7] Wertheimer AM (2014). Aging and cytomegalovirus infection differentially and jointly affect distinct circulating T cell subsets in humans. J. Immunol..

[8] Frasca D, Diaz A, Romero M, Landin AM, Blomberg BB (2011). Age effects on B cells and humoral immunity in humans. Ageing Res. Rev..

[9] Wang C (2014). Effects of aging, cytomegalovirus infection, and EBV infection on human B cell repertoires. J. Immunol..

[10] Almehmadi M, Flanagan BF, Khan N, Alomar S, Christmas SE (2014). Increased numbers and functional activity of CD56^+^ T cells in healthy cytomegalovirus positive subjects. Immunology.

[11] Hassouneh F (2016). Effect of age and latent CMV infection on CD8^+^ CD56^+^ T cells (NKT-like) frequency and functionality. Mech. Ageing Dev..

[12] Chidrawar S (2009). Cytomegalovirus-seropositivity has a profound influence on the magnitude of major lymphoid subsets within healthy individuals. Clin. Exp. Immunol..

[13] Chidrawar SM, Khan N, Chan YL, Nayak L, Moss PA (2006). Ageing is associated with a decline in peripheral blood CD56bright NK cells. Immun. Ageing.

[14] Schulz AR (2015). Low thymic activity and dendritic cell numbers are associated with the immune response to primary viral infection in elderly humans. J. Immunol..

[15] Chong Y (2005). CD27(+) (memory) B cell decrease and apoptosis-resistant CD27(−) (naive) B cell increase in aged humans: implications for age-related peripheral B cell developmental disturbances. Int. Immunol..

[16] Brunner S, Herndler-Brandstetter D, Weinberger B, Grubeck-Loebenstein B (2011). Persistent viral infections and immune aging. Ageing Res. Rev..

[17] Saule P (2006). Accumulation of memory T cells from childhood to old age: Central and effector memory cells in CD4(+) versus effector memory and terminally differentiated memory cells in CD8(+) compartment. Mech. Ageing Dev..

[18] Hutt-Fletcher LM (2007). Epstein–Barr virus entry. J. Virol..

[19] Appay V (2002). Memory CD8^+^ T cells vary in differentiation phenotype in different persistent virus infections. Nat. Med..

[20] Khan N (2004). Herpesvirus-specific CD8 T cell immunity in old age: Cytomegalovirus impairs the response to a coresident EBV infection. J. Immunol..

[21] van den Heuvel D (2016). Cytomegalovirus- and Epstein–Barr virus-induced T-cell expansions in young children do not impair naive T-cell populations or vaccination responses: The generation R study. J. Infect. Dis..

[22] Vescovini R (2004). Different contribution of EBV and CMV infections in very long-term carriers to age-related alterations of CD8^+^ T cells. Exp. Gerontol..

[23] Cevirgel A (2022). Identification of aging-associated immunotypes and immune stability as indicators of post-vaccination immune activation. Aging Cell.

[24] Arruvito L (2014). Identification and clinical relevance of naturally occurring human CD8+HLA-DR+ regulatory T cells. J. Immunol..

[25] Machicote A, Belén S, Baz P, Billordo LA, Fainboim L (2018). Human CD8^+^HLA-DR^+^ regulatory T cells, similarly to classical CD4^+^Foxp3^+^ cells, suppress immune responses via PD-1/PD-L1 axis. Front. Immunol..

[26] Kuijpers TW (2003). Frequencies of circulating cytolytic, CD45RA^+^CD27^−^, CD8^+^ T lymphocytes depend on infection with CMV. J. Immunol..

[27] DeRogatis JM (2023). Cell-intrinsic CD38 expression sustains exhausted CD8^+^ T cells by regulating their survival and metabolism during chronic viral infection. J. Virol..

[28] Ramasubramanian R (2022). Evaluation of T-cell aging-related immune phenotypes in the context of biological aging and multimorbidity in the health and retirement study. Immun. Ageing.

[29] Sayed N (2021). An inflammatory aging clock (iAge) based on deep learning tracks multimorbidity, immunosenescence, frailty and cardiovascular aging. Nat. Aging.

[30] Spielmann G, Johnston CA, O'Connor DP, Foreyt JP, Simpson RJ (2014). Excess body mass is associated with T cell differentiation indicative of immune ageing in children. Clin. Exp. Immunol..

[31] Han JM, Levings MK (2013). Immune regulation in obesity-associated adipose inflammation. J. Immunol..

[32] Pawelec G, Goldeck D, Derhovanessian E (2014). Inflammation, ageing and chronic disease. Curr. Opin. Immunol..

[33] Collins-McMillen D (2019). Alternative promoters drive human cytomegalovirus reactivation from latency. Proc. Natl. Acad. Sci. USA.

[34] Parry HM (2016). Cytomegalovirus viral load within blood increases markedly in healthy people over the age of 70 years. Immun. Ageing.

[35] Stowe RP (2007). Chronic herpesvirus reactivation occurs in aging. Exp. Gerontol..

[36] Aalto SM (1998). Immunoreactivation of Epstein–Barr virus due to cytomegalovirus primary infection. J. Med. Virol..

[37] Lanfermeijer J (2021). Age and CMV-infection jointly affect the EBV-specific CD8^+^ T-cell repertoire. Front. Aging.

[38] van den Berg SPH (2021). Latent CMV infection is associated with lower influenza virus-specific memory T-cell frequencies, but not with an impaired T-cell response to acute influenza virus infection. Front. Immunol..

[39] van den Berg SPH (2018). Negative effect of age, but not of latent cytomegalovirus infection on the antibody response to a novel influenza vaccine strain in healthy adults. Front. Immunol..

[40] Holder B (2010). Epstein–Barr virus but not cytomegalovirus is associated with reduced vaccine antibody responses in Gambian infants. PLoS ONE.

[41] Lasaviciute G (2017). Epstein–Barr virus, but not cytomegalovirus, latency accelerates the decay of childhood measles and rubella vaccine responses—A 10-year follow-up of a Swedish birth cohort. Front. Immunol..

[42] Samson LD (2020). Limited effect of duration of CMV infection on adaptive immunity and frailty: Insights from a 27-year-long longitudinal study. Clin. Transl. Immunol..

[43] Redeker A (2017). The contribution of cytomegalovirus infection to immune senescence is set by the infectious dose. Front. Immunol..

[44] Redeker A, Welten SP, Arens R (2014). Viral inoculum dose impacts memory T-cell inflation. Eur. J. Immunol..

[45] Verberk JDM (2019). Third national biobank for population-based seroprevalence studies in the Netherlands, including the Caribbean Netherlands. BMC Infect. Dis..

[46] Chen S (2012). Cytomegalovirus seropositivity is associated with glucose regulation in the oldest old. Results from the Leiden 85-plus Study. Immun. Ageing.

[47] Pak CY, McArthur RG, Eun HM, Yoon JW (1988). Association of Cytomegalovirus with autoimmune type 1 diabetes. The Lancet.

[48] Alvarez-Lafuente R (2005). Potential relationship between herpes viruses and rheumatoid arthritis: Analysis with quantitative real time polymerase chain reaction. Ann. Rheum. Dis..

[49] Wang H (2017). Cytomegalovirus infection and relative risk of cardiovascular disease (ischemic heart disease, stroke, and cardiovascular death): A meta-analysis of prospective studies up to 2016. J. Am. Heart Assoc..

[50] Samanta M, Harkins L, Klemm K, Britt WJ, Cobbs CS (2003). High prevalence of human cytomegalovirus in prostatic intraepithelial neoplasia and prostatic carcinoma. J. Urol..

[51] van der Heiden M (2016). Differential effects of Cytomegalovirus carriage on the immune phenotype of middle-aged males and females. Sci. Rep..

[52] Sallusto F, Lenig D, Förster R, Lipp M, Lanzavecchia A (1999). Two subsets of memory T lymphocytes with distinct homing potentials and effector functions. Nature.

[53] Appay V, van Lier RA, Sallusto F, Roederer M (2008). Phenotype and function of human T lymphocyte subsets: Consensus and issues. Cytometry A.

[54] Tcherniaeva I, den Hartog G, Berbers G, van der Klis F (2018). The development of a bead-based multiplex immunoassay for the detection of IgG antibodies to CMV and EBV. J. Immunol. Methods.

[55] Samson LD (2020). In-depth immune cellular profiling reveals sex-specific associations with frailty. Immun. Ageing.

[56] Tibshirani R, Walther G, Hastie T (2001). Estimating the number of clusters in a data set via the gap statistic. J. R. Stat. Soc. Ser. B Stat. Methodol..

[57] Hartigan JA, Wong MA (1979). A K-means clustering algorithm. J. R. Stat. Soc. Ser. C Appl. Stat..

