# Supplementary material for: Cytomegalovirus and Epstein–Barr virus co-infected young and middle-aged adults can have an aging-related T-cell phenotype
Source: Sci Rep. 2023 Jul 5;13:10912. doi: 10.1038/s41598-023-37502-5 (PMC10322942; doi:10.1038/s41598-023-37502-5)
Supplement: Supplementary file 1 — Supplementary Information. [file 41598_2023_37502_MOESM1_ESM.docx]

**
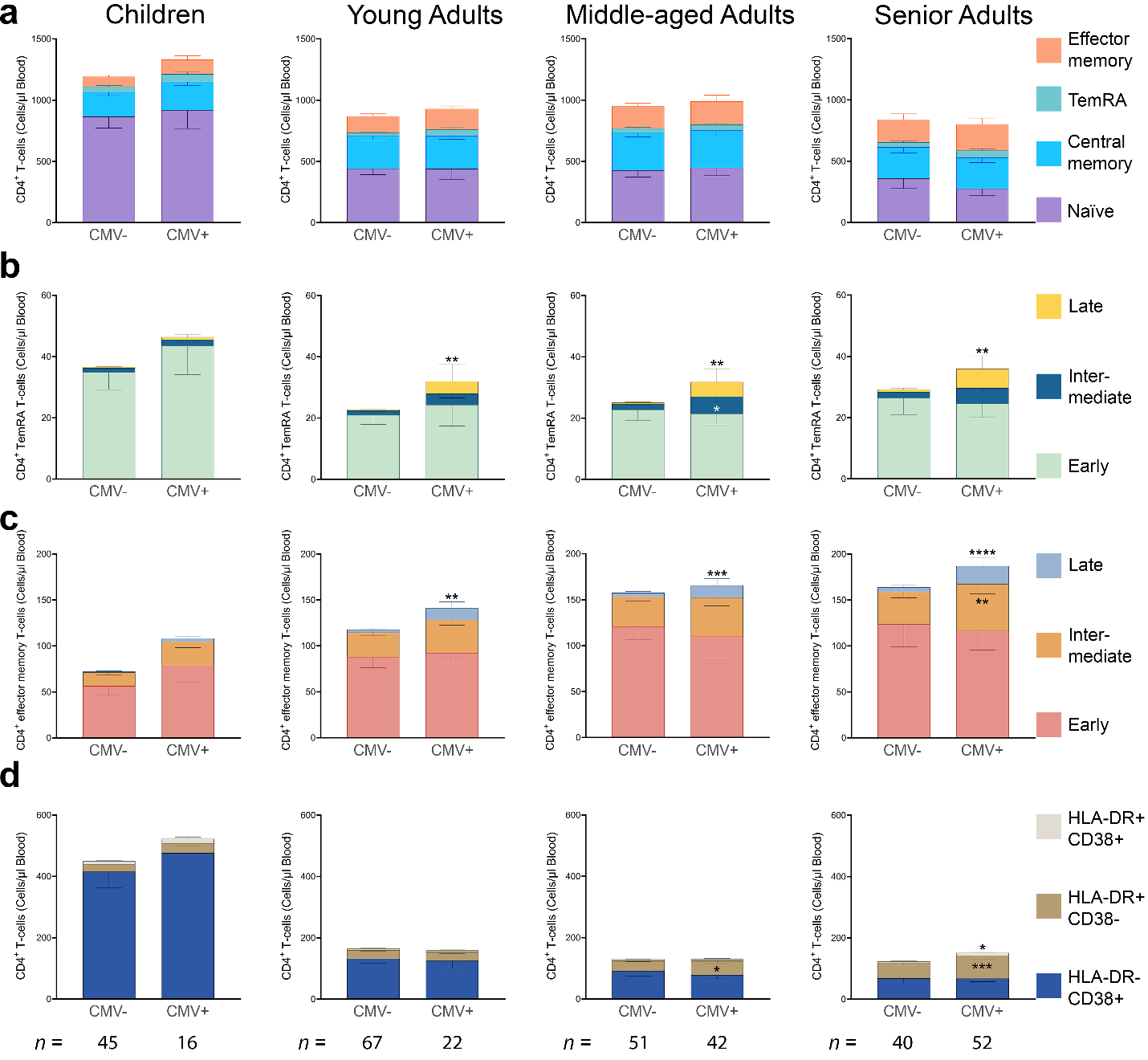
**

**Supplementary Figure S1**, The effect of CMV seropositivity on CD4^+^ T-cells in blood of children and young, middle-aged, and senior adults. **(a)** Numbers of CD4^+^ naïve (CCR7^+^ CD45RA^+^), central memory (CCR7^+^ CD45RA^-^), effector memory (CCR7^-^ CD45RA^-^), or TemRA (CCR7^-^ CD45RA^+^) T-cells in CMV-seropositive or CMV-seronegative individuals. The differentiation status of **(b)** CD4^+^ TemRA T-cells and **(c)** CD4^+^ effector memory T-cells was determined and the cells were further specified into early- (CD45RA^+^ CD28^+^ CD27^+^), intermediate- (CD45RA^+^ CD28^+^ CD27^-^), or late-differentiated (CD45RA^+^ CD28^-^ CD27^-^). **(d)** Differences in HLA-DR^+/-^ and CD38^+/-^ CD4^+^ T-cell numbers between CMV-seropositive or CMV-seronegative individuals are depicted. Cell numbers are per µl blood and data are geomeans with 95% confidence intervals. **p* < 0.05, ***p* < 0.01, ****p* < 0.001, and *****p* < 0.0001. A two-way ANOVA with a Dunnett’s multiple comparison test was used as statistical test. *n* = 61 for children, *n* = 89 for young adults, *n* = 93 for middle-aged adults, and *n* = 92 for senior adults. Number of CMV^-^ or CMV^+^ individuals per age group are indicated within the figure.

**
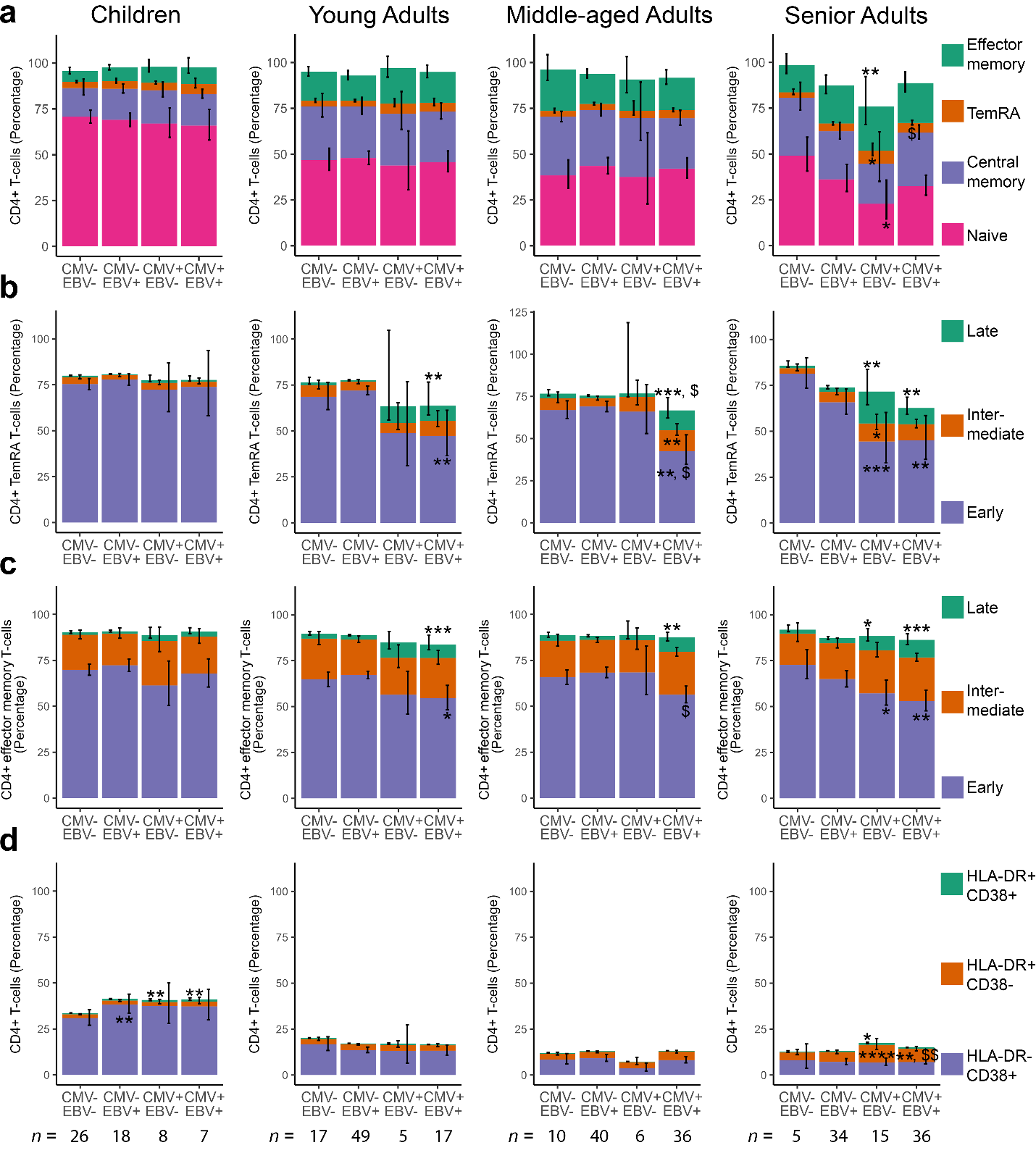
**

**Supplementary Figure S2**, CD4^+^ T-cell percentages in blood of CMV^-^ EBV^-^, CMV^-^ EBV^+^, CMV^+^ EBV^-^, or CMV^+^ EBV^+^ children and young, middle-aged, and senior adults. For these four CMV and EBV seropositivity depending groups, **(a)** percentages of CD4^+^ naïve (CCR7^+^ CD45RA^+^), central memory (CCR7^+^ CD45RA^-^), effector memory (CCR7^-^ CD45RA^-^), or TemRA (CCR7^-^ CD45RA^+^) T-cells are shown (gated from all CD4^+^ T-cells), together with percentages of early- (CD45RA^+^ CD28^+^ CD27^+^), intermediate- (CD45RA^+^ CD28^+^ CD27^-^), or late-differentiated (CD45RA^+^ CD28^-^ CD27^-^) **(b)** CD4^+^ TemRA T-cells (gated from CD4^+^ TemRA T-cells) or **(c)** CD8^+^ effector memory T-cells (gated from CD4^+^ effector memory T-cells). **(d)** Differences in HLA-DR^+/-^ and CD38^+/-^ CD4^+^ T-cell percentages (gated from all CD4^+^ T-cells) from all between CMV^-^ EBV^-^, CMV^-^ EBV^+^, CMV^+^ EBV^-^, or CMV^+^ EBV^+^ individuals. Data are geomeans with 95% confidence intervals. A two-way ANOVA and Holm-Bonferroni post-hoc test was used as statistical test. * ^or $^*p* < 0.05, ***p* < 0.01, ****p* < 0.001, and *****p* < 0.0001. An asterisk was used to indicate significant differences compared to the CMV^-^ EBV^-^, non-infected group and a dollar sign indicates a comparison with the CMV^+^ EBV^-^ group. *n* = 59 for children, *n* = 88 for young adults, *n* = 92 for middle-aged adults, and *n* = 90 for senior adults. Number of CMV^-^ EBV^-^, CMV^-^ EBV^+^, CMV^+^ EBV^-^, or CMV^+^ EBV^+^ individuals per age group are indicated within the figure.

**
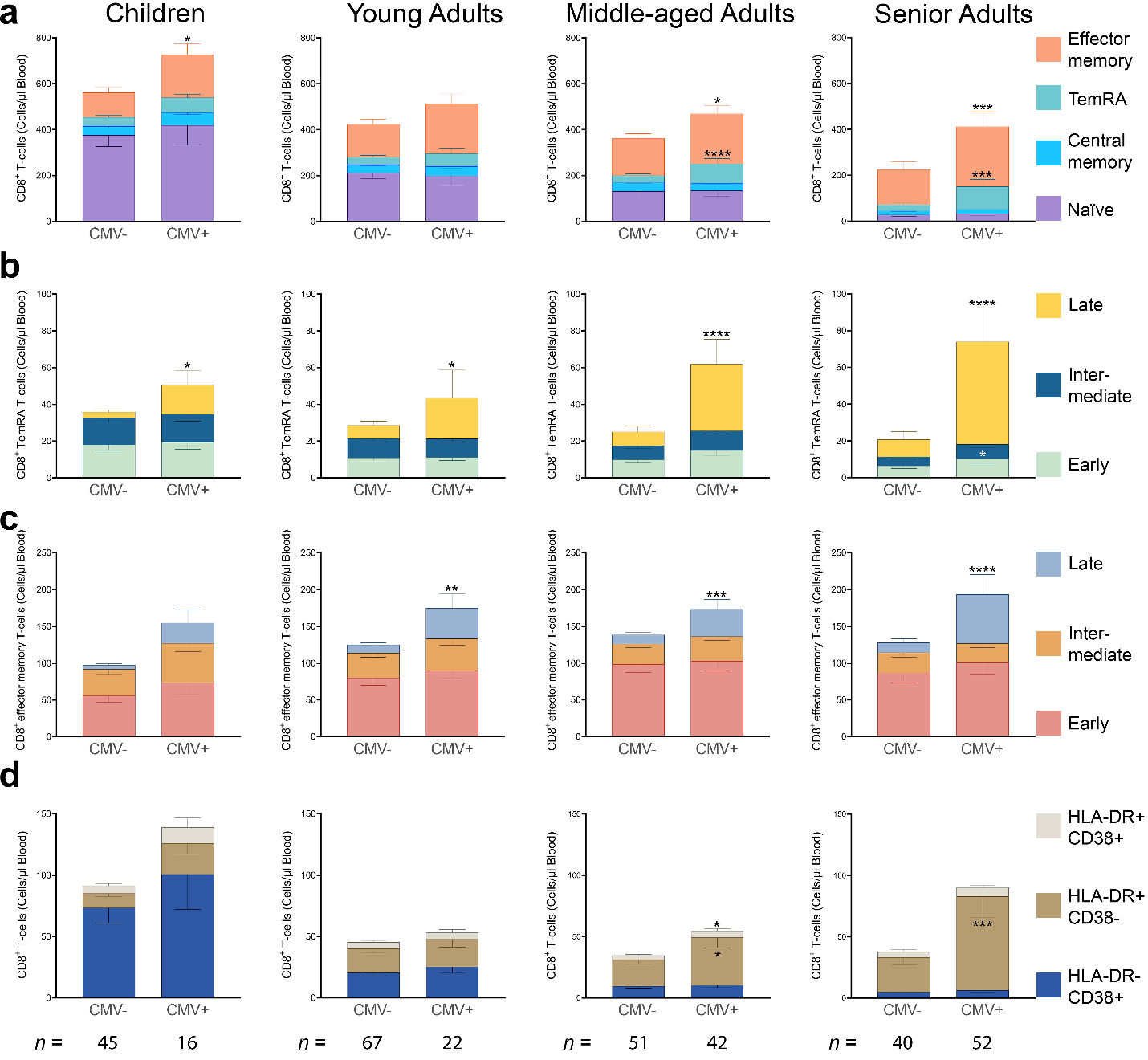
**

**Supplementary Figure S3**, The effect of CMV seropositivity on CD8^+^ T-cells in blood of children and young, middle-aged, and senior adults. **(a)** Numbers of CD8^+^ naïve (CCR7^+^ CD45RA^+^), central memory (CCR7^+^ CD45RA^-^), effector memory (CCR7^-^ CD45RA^-^), or TemRA (CCR7^-^ CD45RA^+^) T-cells in CMV-seropositive or CMV-seronegative individuals. Numbers of early- (CD45RA^+^ CD28^+^ CD27^+^), intermediate- (CD45RA^+^ CD28^-^ CD27^+^), or late-differentiated (CD45RA^+^ CD28^-^ CD27^-^) **(b)** CD8^+^ TemRA T-cells or **(c)** CD8^+^ effector memory T-cells for CMV^+^ or CMV^-^ individuals. **(d)** HLA-DR^+/-^ and CD38^+/-^ CD8^+^ T-cell numbers for CMV-seropositive or CMV-seronegative individuals. Cell numbers are per µl blood and data are geomeans with 95% confidence intervals. **p* < 0.05, ***p* < 0.01, ****p* < 0.001, and *****p* < 0.0001. A two-way ANOVA with a Dunnett’s multiple comparison test was used as statistical test. *n* = 61 for children, *n* = 89 for young adults, *n* = 93 for middle-aged adults, and *n* = 92 for senior adults. Number of CMV^-^ or CMV^+^ individuals per age group are indicated within the figure.

**
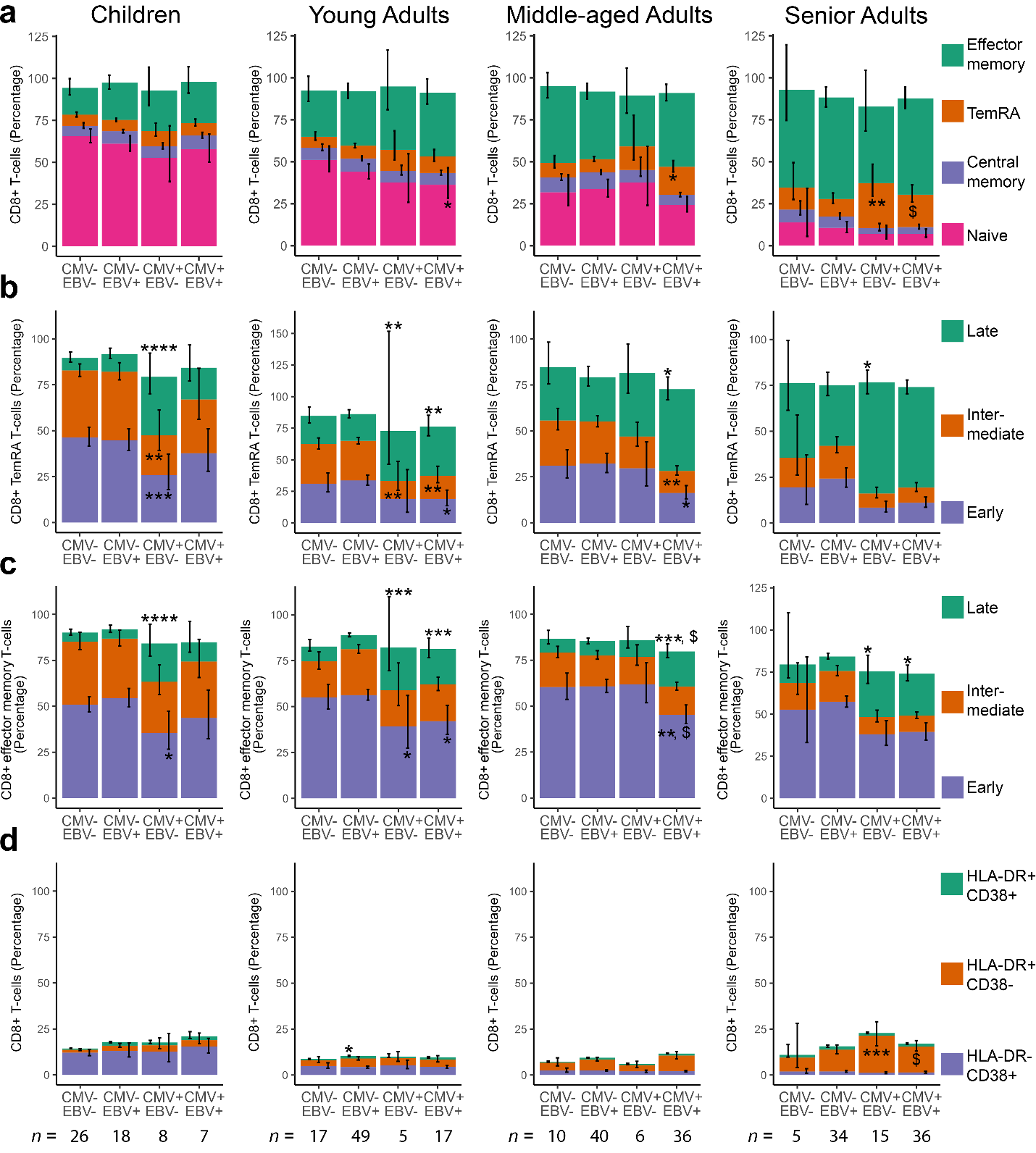
**

**Supplementary Figure S4**, CD8^+^ T-cell percentages in blood of CMV^-^ EBV^-^, CMV^-^ EBV^+^, CMV^+^ EBV^-^, or CMV^+^ EBV^+^ children and young, middle-aged, and senior adults. **(a)** Percentages of CD8^+^ naïve (CCR7^+^ CD45RA^+^), central memory (CCR7^+^ CD45RA^-^), effector memory (CCR7^-^ CD45RA^-^), or TemRA (CCR7^-^ CD45RA^+^) T-cells from all CD8^+^ T-cells in CMV^-^ EBV^-^, CMV^-^ EBV^+^, CMV^+^ EBV^-^, or CMV^+^ EBV^+^ individuals. For these four CMV and EBV seropositivity depending groups, percentages of early- (CD45RA^+^ CD28^+^ CD27^+^), intermediate- (CD45RA^+^ CD28^-^ CD27^+^), or late-differentiated (CD45RA^+^ CD28^-^ CD27^-^) **(b)** CD8^+^ TemRA T-cells (gated from all CD8^+^ TemRA T-cells) or **(c)** CD8^+^ effector memory T-cells (gated from CD8^+^ effector memory T-cells) are shown. **(d)** Percentages of HLA-DR^+/-^ and CD38^+/-^ CD8^+^ T-cell numbers were determined from all CD8^+^ T-cells. Data are geomeans with 95% confidence intervals. * ^or $^*p* < 0.05, ** *p* < 0.01, ****p* < 0.001, and *****p* < 0.0001. A two-way ANOVA and Holm-Bonferroni post-hoc test was used as statistical test. An asterisk was used to indicate significant differences compared to the CMV^-^ EBV-, non-infected group and a dollar sign indicates a comparison with the CMV^+^ EBV^-^ group. *n* = 59 for children, *n* = 88 for young adults, *n* = 92 for middle-aged adults, and *n* = 90 for senior adults. Number of CMV^-^ EBV^-^, CMV^-^ EBV^+^, CMV^+^ EBV^-^, or CMV^+^ EBV^+^ individuals per age group are indicated within the figure.

**
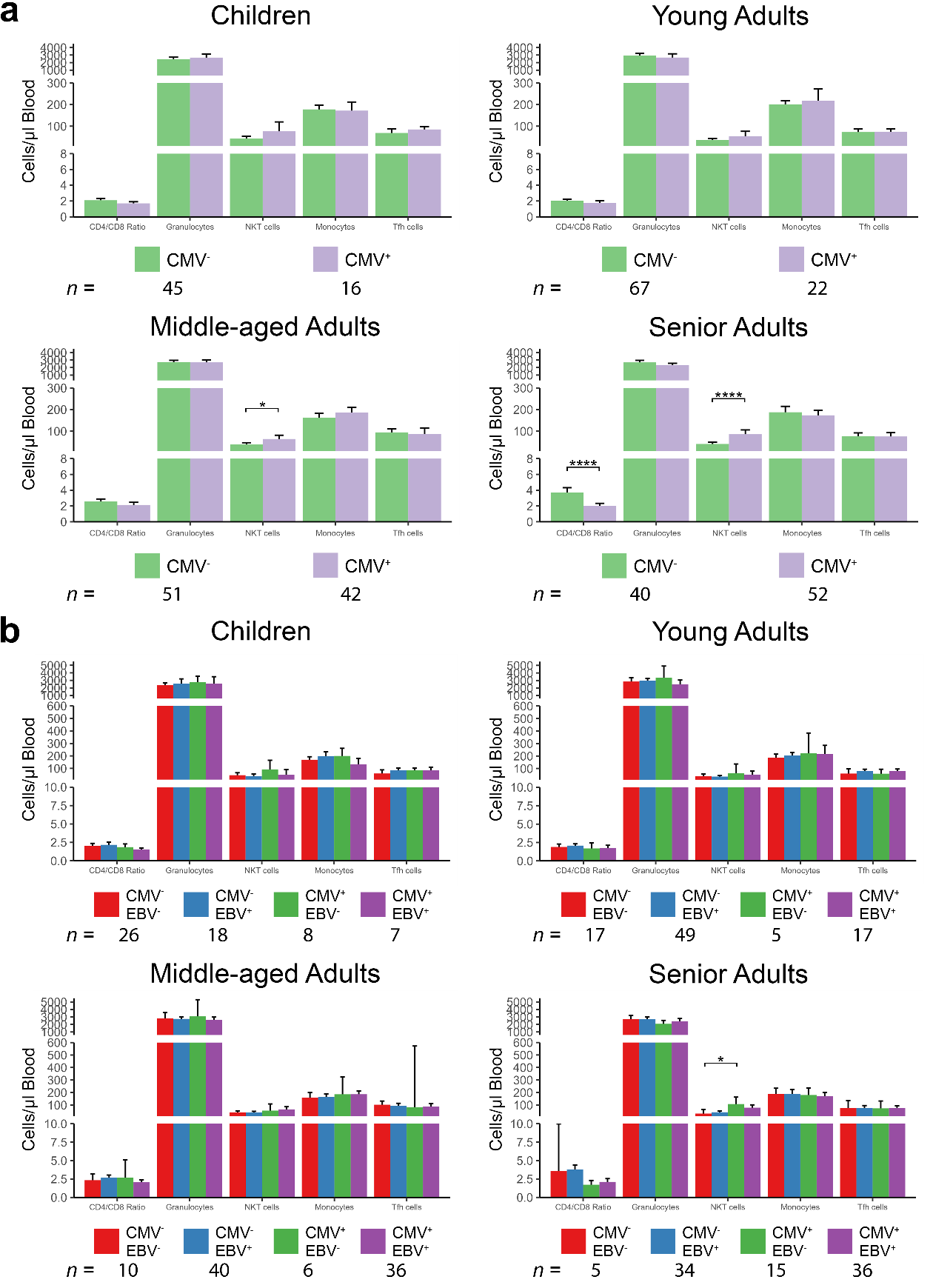
**

**Supplementary Figure S5**, **(a)** CD4/CD8 ratio’s and numbers of granulocytes (CD45^+^ SSC^high^), NKT cells (CD3^+^ CD56^+^), monocytes (CD45^+^ SSC^middle^), and Tfh cells (CD4^+^ CD45RA^-^ and CXCR5^+^) for CMV-seronegative or CMV-seropositive children (*n* = 61) and young (*n* = 89), middle-aged (*n* = 93), and senior adults (*n* = 92). Number of CMV^-^ or CMV^+^ individuals per age group are indicated within the figure. **(b)** CD4/CD8 ratio’s and numbers of granulocytes, NKT cells, monocytes, and Tfh cells for CMV^-^ EBV^-^, CMV^-^ EBV^+^, CMV^+^ EBV^-^, or CMV^+^ EBV^+^ children (*n* = 59) and young (*n* = 88), middle-aged (*n* = 92), and senior adults (*n* = 90). Number of CMV^-^ EBV^-^, CMV^-^ EBV^+^, CMV^+^ EBV^-^, or CMV^+^ EBV^+^ individuals per age group are indicated within the figure. Cell numbers are per µl blood and data are geomeans with 95% confidence intervals. **p* < 0.05, ***p* < 0.01, and *****p* < 0.0001. The statistical test used was a two-way ANOVA with a Dunnett’s multiple comparison test.

**
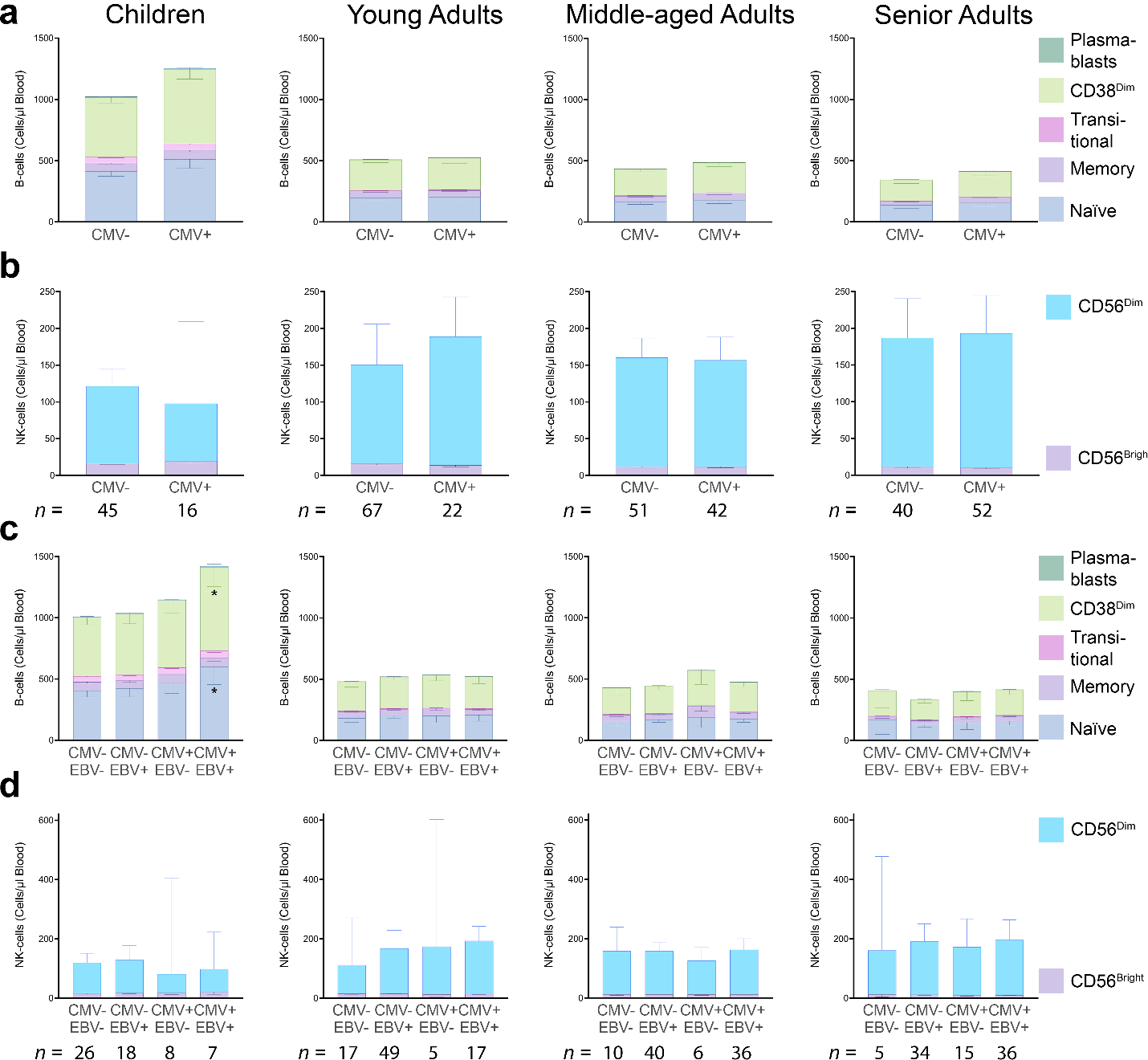
**

**Supplementary Figure S6**, Numbers of **(a)** naïve (CD19^+^ CD27^-^), memory (CD19^+^ CD27^+^), transitional (CD19^+^ CD38^Bright^ CD27^-^), CD38^Dim^ B-cells, and plasmablasts (CD19^+^ CD38^Bright^ CD27^+^) or **(b)** CD56^Bright^ (CD16^Dim/-^) and CD56^Dim^ (CD16^Bright^) NK-cells for CMV^-^ and CMV^+^ (*n* = 61) and young (*n* = 89), middle-aged (*n* = 93), and senior adults (*n* = 92). Number of CMV^-^ or CMV^+^ individuals per age group are indicated within the figure. Numbers of **(c)** naïve, memory, transitional, CD38^Dim^ B-cells, and plasmablasts or **(d)** CD56^Bright^ and CD56^Dim^ NK-cells for CMV^-^ EBV^-^, CMV^-^ EBV^+^, CMV^+^ EBV^-^, or CMV^+^ EBV^+^ children (*n* = 59) and young (*n* = 88), middle-aged (*n* = 92), and senior adults (*n* = 90). Number of CMV^-^ EBV^-^, CMV^-^ EBV^+^, CMV^+^ EBV^-^, or CMV^+^ EBV^+^ individuals per age group are indicated within the figure. Cell numbers are per µl blood and data are geomeans with 95% confidence intervals. **p* < 0.05. The statistical test used was a two-way ANOVA with a Dunnett’s multiple comparison test.

**
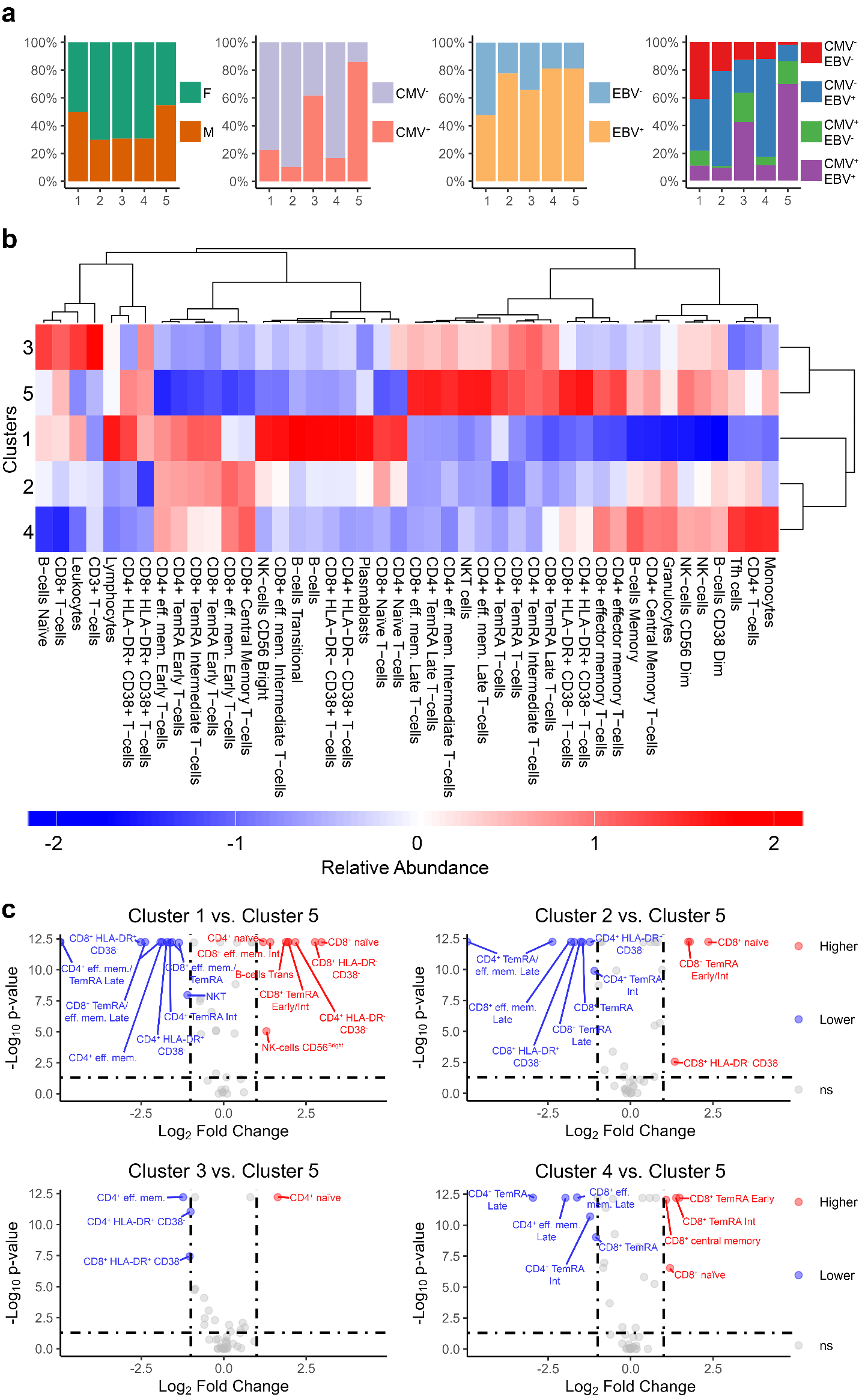
**

**Supplementary Figure S7**, Additional information on the five clusters identified with a gap statistical analysis. **(a)** Percentage of male vs female, CMV^-^ vs CMV^+^, EBV^-^ vs EBV^+^ per cluster or the percentage of individuals that are CMV^-^ EBV^-^, CMV^-^ EBV^+^, CMV^+^ EBV^-^, or CMV^+^ EBV^+^ per cluster. **(b)** Heatmap with the relative abundance (ranging from 2 to -2) of the 44 immune cell subsets used for the cluster analysis per cluster, with dendrograms that show the hierarchical relationship between the cluster and the immune cells. **(c)** Volcano plots depicting log10-transformed *p*-values and log2-transformed fold changes per immune cells comparing cluster-5 with cluster-1, -2, -3, and -4. In blue are the immune cells that had significantly lower numbers and a relevant fold change compared to cluster-5, whereas immune cells in red had significantly higher numbers and a relevant fold change compared to cluster-5. A log2-transformed fold change of 1 (horizontal dashed line) and a log10-transformed *p*-value of 0.05 (1.301; vertical dashed line) were considered relevant and significant, respectively. The statistical test used was a one-way ANOVA with a TukeyHSD post-hoc test. *n* = 68 for cluster-1, *n* = 80 for cluster-2, *n* = 40 for cluster-3, *n* = 65 for cluster-4, and *n* = 82 for cluster-5.

**
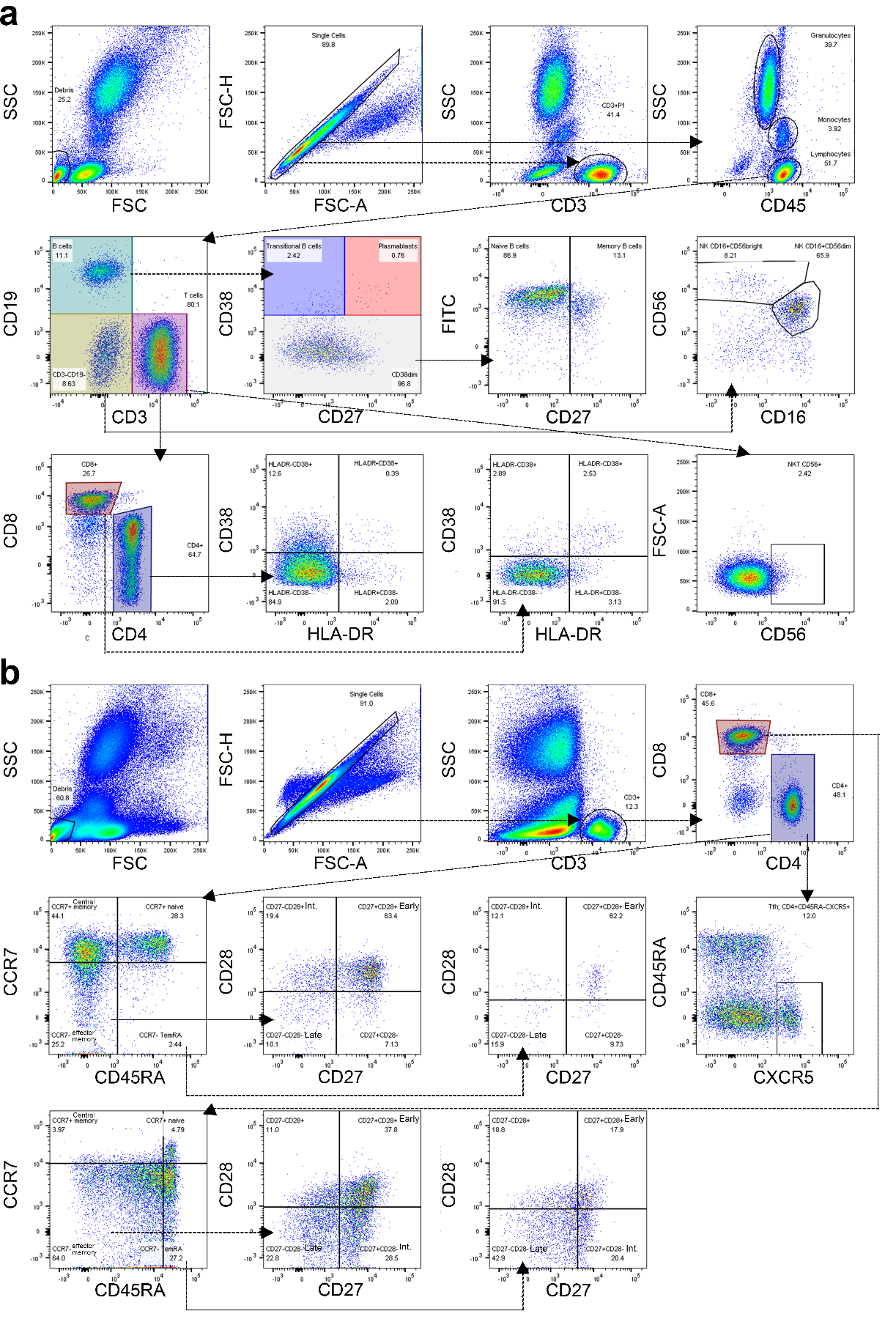
**

**Supplementary Figure S8**, The gating strategy used for **(a)** antibody panel 1 and **(b)** antibody panel 2.

**Supplementary Table S1**, Absolute leukocyte and lymphocyte cell numbers per age group, independent of CMV- and EBV-serostatus. The reported values are geometric means of absolute cell numbers, number of cells/µl blood, with 95% confidence intervals. Cell counts that were significantly different are written in bold and in superscript it is indicated whether the value was significantly different from the 4-8 years (a), 18-25 years (b), or 39-45 years (c) age group. * p < 0.05, ** p < 0.01, *** p < 0.001, and **** p < 0.0001.

|  | Children  4-8 years | Young Adults  18-25 years | Middle-aged Adults  39-45 years | Senior Adults  64-70 years |
| --- | --- | --- | --- | --- |
|  | Leukocyte counts (geometric mean [95% CI]) | | | |
| Leukocytes | 5807 [5447 – 6191] | 5412 [5133 – 5706] | 5107 [4828 – 5402] | **4837 [4583 – 5106] ***^a^** |
| Lymphocytes | 3038 [2825 – 3268] | **2213 [2088 – 2346] ****^a^** | **2139 [2012 – 2273] ****^a^** | **2053 [1910 – 2207] ****^a^** |
| CD3^+^ T-cells | 2178 [2016 – 2354] | **1623 [1523 – 1728] ****^a^** | **1621 [1523 – 1724] ****^a^** | **1463 [1357 – 1578] ****^a^** |
| CD4^+^ T-cells | 1267 [1172 – 1370] | **945.8 [877.5 – 1020] ****^a^** | **1041 [967– 1120] **^a^** | **950.9 [878.9 – 1029] ****^a^** |
| Naïve | 877.5 [799.6 – 963] | **444 [399.6 – 493.2] ****^a^** | **436.6 [394 – 483.7] ****^a^** | **309.9 [261.4 – 367.3] ****^a^** |
| Central Memory | 206.1 [179.9 – 236.2] | **267.2 [239 – 298.8] ***^a^** | **307.5 [277.1 – 341.2] ****^a^** | **254.3 [222.6 – 290.6] *^a^** |
| TemRA | 51 [44.1 – 58.9] | **33.7 [28.8 – 39.6]** *****^a^** | **39.1 [33.6 – 45.4] *^a^** | 47.9 [40.5 – 56.6] |
| Early | 38.2 [32.9 – 44.4] | **21.7 [18.8 – 25] ***^a^** | **22.2 [19.6 – 25.2] ****^a^** | **25.35 [21.8 – 29.4] **^a^** |
| Intermediate | 1.6 [1.3 – 1.9] | 1.9 [1.5 – 2.4] | **2.9 [2.3 – 3.8]** ****^a^** | **3.4 [2.6 – 4.4] ***^a,^ **^b^** |
| Late | 0.4 [0.3 – 0.6] | 0.7 [0.4 – 1] | **1.5 [1 – 2.3]** ******^a,^ *^b^** | **2.6 [1.8 – 3.9] ****^a,^ ****^b^** |
| Effector Memory | 89.5 [76.7 – 104.4] | **141.7 [126.6 – 158.6] ****^a^** | **183.6 [161.5 – 208.8] ^a^****^,^ *^b^** | **202.6 [174.7 – 235] ****^a,^ ****^b^** |
| Early | 61.9 [52.8 – 72.6] | **89.7 [80.2 – 100.2] **^a^** | **116 [102.3 – 131.5] ****^a,^ **^b^** | **119.7 [103.4 – 138.4] ] ****^a,^ ***^b^** |
| Intermediate | 17.2 [14.6 – 20.3] | **28.3 [25.5 – 32.7]** ******^a^** | **36.7 [31.8 – 42.3] ****^a^** | **43.5 [36.8 – 51.5] ****^a,^ ***^b^** |
| Late | 1.6 [1.2 – 2] | **4.6 [3.6 – 5.7]** ******^a^** | **6.9 [5.2 – 9.1] ****^a^** | **10.7 [7.9 – 14.4] ****^a,^ ***^b^** |
| HLA-DR^+^ CD38^+^ | 10.8 [9.4 – 12.4] | **5.3 [4.7 – 6] ****^a^** | **5 [4.5 – 5.6] ****^a^** | **7.2 [6.4 – 8.2] **^a,^ **^b,^ ***^c^** |
| HLA-DR^+^ CD38^-^ | 26.4 [23.4 – 29.8] | 28.3 [25.3 – 31.6] | **39.6 [35.6 – 44] ****^a,^ **^b^** | **62.7 [55.7 – 70.5] ****^a,^ ****^b,^ ****^c^** |
| HLA-DR^-^ CD38^+^ | 435.4 [391.9 – 483.8] | **132.1 [118.8 – 147] ****^a^** | **85 [73.9 – 97.9] ****^a,^ ***^b^** | **67.8 [58.7 – 78.4] ****^a,^ ****^b^** |
| CD8^+^ T-cells | 641.2 [579.8 – 709.1] | **484.5 [448.1 – 523.8] **^a^** | **445.3 [412.3 – 481] ****^a^** | **368.1 [323.7 – 418.6] ****^a,^ **^b^** |
| Naïve | 395.4 [349.8 – 446.8] | **208.1 [187.1 – 231.5] ****^a^** | **133.2 [117.2 – 151.2] ****^a,^ **^b^** | **31 [25.2 – 37.6] ****^a,^ ****^b,^ ****^c^** |
| Central Memory | 43.7 [37.6 – 50.7] | 37 [32.5 – 42] | 35 [30.8 – 39.6] | **17.7 [14.5 – 21.7] ****^a,^ ****^b,^ ****^c^** |
| TemRA | 45.3 [39.3 – 52.2] | 38.5 [32.3 – 45.8] | 49 [40.8 – 58.7] | **58.6 [46.3 – 74.2] **^b^** |
| Early | 18.6 [16.2 – 21.4] | **11 [9.6 – 12.7] ****^a^** | **12 [10.5 – 13.8] ***^a^** | **8.5 [7 – 10.2] ****^a,^ *^c^** |
| Intermediate | 15.4 [13.1 – 18] | **10.4 [8.9 – 12.2]** ***^a^** | **8.7 [7.6 – 9.9]** ******^a^** | **6.5 [5.2 – 8.1]** ******^a,^ **^b^** |
| Late | 4.7 [3.5 – 6.4] | **9.6 [7.4 – 12.4]** ***^a^** | **15.6 [11.8 – 20.6] ****^a^** | **26.7 [19.9 – 35.8] ****^a,^ ****^b^** |
| Effector Memory | 124.9 [106.7 – 146.2] | 159.4 [141.2 – 180] | **184.7 [167.3 – 204] ***^a^** | **205.8 [175.9 – 240.9] ****^a,^ *^b^** |
| Early | 60.2 [52.1 – 69.6] | **82.5 [74 – 92] **^a^** | **100.3 [91.8 – 109.7] ****^a^** | **94.6 [83.4 – 107.3] ****^a^** |
| Intermediate | 41.2 [34.4 – 49.2] | 36 [30.9 – 42.1] | **30.3 [26.5 – 34.7]** ***^a^** | **26 [21.7 – 31.2]** *****^a,^ *^b^** |
| Late | 8.1 [5.8 – 11.2] | **15.8 [12.7 – 19.6] *^a^** | **20.3 [16.4 – 25.1] ***^a^** | **33.2 [25 – 44.1] ****^a,^ **^b^** |
| HLA-DR^+^ CD38^+^ | 7.8 [6.3 – 9.6] | 5.3 [4.4 – 6.4] | **4.1 [3.5 – 4.8] ***^a^** | **6.1 [5.1 – 7.4] *^c^** |
| HLA-DR^+^ CD38^-^ | 14.7 [11.8 – 18.3] | 20.2 [16.9 – 24.1] | **28.4 [24.2 – 33.4]** *****^a^** | **50.4 [41.1 – 61.8]** ******^a,^ ****^b,^ **^c^** |
| HLA-DR^-^ CD38^+^ | 82.1 [69.8 – 96.6] | **21.8 [19.3 – 24.7] ****^a^** | **10 [8.7 – 11.6] ****^a,^ ****^b^** | **5.8 [4.9 – 6.8] ****^a,^ ****^b,^ **^c^** |
| CD4^+^/8^+^ T cell ratio | 2.1 [1.8 – 2.2] | 2 [1.8 – 2.1] | **2.4 [2.2 – 2.6] *^a,^ *^b^** | **2.6 [2.3 – 3] **^a,^ ***^b^** |
| T follicular helper cells | 73 [61.1 – 87.1] | 73.2 [63.8 – 84] | **91.6 [79.2 – 105.9] *^a,^ **^b^** | 75.6 [65.6 – 87.1] |
| NKT cells | 48.8 [39.3 – 60.7] | 39.5 [33.8 – 46.2] | 49.2 [42.5 – 57.1] | **62 [52.6 – 73] **^b^** |
| NK-cells | 122.1 [103.6 – 143.9] | **177.1 [153 – 205] ***^a^** | **161.3 [143.7 – 181.1]** ***^a^** | **196.6 [168.8 – 229] ****^a,^ *^c^** |
| NK-cells CD56^Dim^ | 94.5 [73.3 – 121.9] | **144.4 [111.1 – 187.5] *^a^** | 146.5 [129.5 – 165.8] | **179.4 [150.4 – 214] ****^a,^ *^c^** |
| NK-cells CD56^Bright^ | 17.6 [15.6 – 20] | 15.4 [14.1 – 16.9] | **12.4 [11.1 – 13.8] ***^a,^ *^b^** | **10.9 [9.7 – 12.3] ****^a,^ ***^b^** |
| B-cells | 579.2 [533.8 – 628.4] | **268.1 [286.8 – 291.8] ****^a^** | **239.8 [218.8 – 262.9] ****^a^** | **198.7 [173.1 – 228] ****^a^** |
| Naïve | 440 [403.6 – 479.7] | **201.3 [183.5 – 220.9] ****^a^** | **170.9 [154.2 – 189.4] ****^a^** | **148.1 [127.8 – 171.8] ****^a^** |
| Memory | 71 [63.1 – 80] | **48.7 [44.2 – 53.7] ****^a^** | **51 [45 – 58] **^a^** | **36.8 [31.9 – 42.4] ****^a,^ **^c^** |
| Transitional | 49.4 [44.4 – 55] | **8.2 [7 – 9.6] ****^a^** | **5.8 [5 – 6.8] ****^a^** | **5 [4 – 6] ****^a,^ *^b^** |
| CD38^Dim^ | 517.1 [475.4 – 562.5] | **254.2 [233.2 – 277.1] ****^a^** | **230.6 [210.3 – 252.9] ****^a^** | **190.8 [166.2 – 219] ****^a^** |
| Plasmablasts | 6.3 [5.3 – 7.6] | **2.4 [2 – 2.8] ****^a^** | **1.5 [1.3 – 1.8] ****^a,^ **^b^** | **1.4 [1.1 – 1.7] ****^a,^ ***^b^** |
| Granulocytes | 2483 [2272 – 2714] | 2881 [2672 – 3107] | 2702 [2511 – 2907] | 2465 [2286 – 2657] |
| Monocytes | 176.7 [161.5 – 193.3] | 204.3 [187.8 – 222.3] | 173.3 [159.7 – 188.1] | 179.6 [163.9 – 196.8] |

**Supplementary Table S2**, A list of the antibodies used within this study to immunophenotype the heparin blood of the study participants. The antibodies were grouped into two different flow panels (†Cytognos, S.L., Salamanca, Spain; ‡Beckman Coulter, Fullerton, CA, USA; §Biolegend, San Diego, CA, USA; ¶R&D Systems, Minneapolis, MN, USA).

| Antigen | Fluorochrome | Clone | Company | Catalogue # |  |
| --- | --- | --- | --- | --- | --- |
|  | Panel 1 (Trucount tube) | | | | |
| CD45 | OC515 | GA90 | Cytognos† | CYT-450C |  |
| CD3 | BV711 | UCHT1 | BD Biosciences | 563725 |  |
| CD4 | PerCP-Cy5.5 | SK3 | BD Biosciences | 332772 |  |
| CD8 | FITC | SK1 | BD Biosciences | 345772 |  |
| HLA-DR | PE-CF594 | G46-6 | BD Biosciences | 562304 |  |
| CD38 | APC-H7 | HB7 | BD Biosciences | 656646 |  |
| CD19 | PE-Cy7 | J3-119 | Beckman Coulter‡ | IM3628 |  |
| CD27 | BV421 | M-T271 | Biolegend§ | 356418 |  |
| CD56 | APC | B159 | BD Biosciences | 555518 |  |
| CD16 | PE | B73.1 | BD Biosciences | 332779 |  |
|  | Panel 2 (normal Falcon tube) | | | | |
| CD3 | BV711 | UCHT1 | BD Biosciences | 563725 |  |
| CD4 | BV510 | RPA-T4 | Biolegend | 300546 |  |
| CD8 | APC-H7 | SK1 | BD Biosciences | 560179 |  |
| CCR7 | PE-CF594 | 150503 | BD Biosciences | 562381 |  |
| CD45RA | BV650 | HI100 | Biolegend | 304136 |  |
| CD27 | BV421 | M-T271 | Biolegend | 356418 |  |
| CD28 | PerCP-Cy5.5 | CD28.2 | BD Biosciences | 560685 |  |
| CXCR5 | APC | 51505 | R&D Systems¶ | FAB190A-100 |  |

**Supplementary Table S3**, A list of all immune cells and there expression markers quantified in blood of the study participants with flow cytometry.

| Immune cell type | Markers |
| --- | --- |
| Leukocytes | CD45^+^ SSC^middle/high^ |
| Lymphocytes | CD45^+^ SSC^low^ |
| T-cells | CD3^+^ |
| CD4^+^ T-cells | CD3^+^ CD4^+^ |
| CD4^+^ Naïve T-cells | CD3^+^ CD4^+^ CCR7^+^ CD45RA^+^ |
| CD4^+^ Central Memory T-cells | CD3^+^ CD4^+^ CCR7^+^ CD45RA^-^ |
| CD4^+^ TemRA T-cells | CD3^+^ CD4^+^ CCR7^-^ CD45RA^+^ |
| CD4^+^ TemRA Early T-cells | CD3^+^ CD4^+^ CCR7^-^ CD45RA^+^ CD28^+^ CD27^+^ |
| CD4^+^ TemRA Intermediate T-cells | CD3^+^ CD4^+^ CCR7^-^ CD45RA^+^ CD28^+^ CD27^-^ |
| CD4^+^ TemRA Late T-cells | CD3^+^ CD4^+^ CCR7^-^ CD45RA^+^ CD28^-^ CD27^-^ |
| CD4^+^ Effector Memory T-cells | CD3^+^ CD4^+^ CCR7^-^ CD45RA^-^ |
| CD4^+^ Effector Memory Early T-cells | CD3^+^ CD4^+^ CCR7^-^ CD45RA^-^ CD28^+^ CD27^+^ |
| CD4^+^ Effector Memory Intermediate T-cells | CD3^+^ CD4^+^ CCR7^-^ CD45RA^-^ CD28^+^ CD27^-^ |
| CD4^+^ Effector Memory Late T-cells | CD3^+^ CD4^+^ CCR7^-^ CD45RA^-^ CD28^-^ CD27^-^ |
| CD4^+^ HLA-DR^+^ CD38^+^ T-cells | CD4^+^ HLA-DR^+^ CD38^+^ |
| CD4^+^ HLA-DR^+^ CD38^-^ T-cells | CD4^+^ HLA-DR^+^ CD38^-^ |
| CD4^+^ HLA-DR^-^ CD38^+^ T-cells | CD4^+^ HLA-DR^-^ CD38^+^ |
| CD8^+^ T-cells | CD3^+^ CD8^+^ |
| CD8^+^ Naïve T-cells | CD3^+^ CD8^+^ CCR7^+^ CD45RA^+^ |
| CD8^+^ Central Memory T-cells | CD3^+^ CD8^+^ CCR7^+^ CD45RA^-^ |
| CD8^+^ TemRA T-cells | CD3^+^ CD8^+^ CCR7^-^ CD45RA^+^ |
| CD8^+^ TemRA Early T-cells | CD3^+^ CD8^+^ CCR7^-^ CD45RA^+^ CD28^+^ CD27^+^ |
| CD8^+^ TemRA Intermediate T-cells | CD3^+^ CD8^+^ CCR7^-^ CD45RA^+^ CD28^-^ CD27^+^ |
| CD8^+^ TemRA Late T-cells | CD3^+^ CD8^+^ CCR7^-^ CD45RA^+^ CD28^-^ CD27^-^ |
| CD8^+^ Effector Memory T-cells | CD3^+^ CD8^+^ CCR7^-^ CD45RA^-^ |
| CD8^+^ Effector Memory Early T-cells | CD3^+^ CD8^+^ CCR7^-^ CD45RA^-^ CD28^+^ CD27^+^ |
| CD8^+^ Effector Memory Intermediate T-cells | CD3^+^ CD8^+^ CCR7^-^ CD45RA^-^ CD28^-^ CD27^+^ |
| CD8^+^ Effector Memory Late T-cells | CD3^+^ CD8^+^ CCR7^-^ CD45RA^-^ CD28^-^ CD27^-^ |
| CD8^+^ HLA-DR^+^ CD38^+^ T-cells | CD8^+^ HLA-DR^+^ CD38^+^ |
| CD8^+^ HLA-DR^+^ CD38^-^ T-cells | CD8^+^ HLA-DR^+^ CD38^-^ |
| CD8^+^ HLA-DR^-^ CD38^+^ T-cells | CD8^+^ HLA-DR^-^ CD38^+^ |
| Tfh cells | CD4^+^ CD45RA^-^ and CXCR5^+^ |
| NKT cells | CD3^+^ CD56^+^ |
| NK-cells | CD16^Dim/-^ CD56^Bright^ and CD16^Bright^ CD56^Dim^ |
| NK-cells CD56 Bright | CD16^Dim/-^ CD56^Bright^ |
| NK-cells CD56 Dim | CD16^Bright^ CD56^Dim^ |
| B-cells | CD19^+^ |
| B-cells Naïve | CD19^+^ CD27^-^ |
| B-cells Memory | CD19^+^ CD27^+^ |
| B-cells Transitional | CD19^+^ CD38^Bright^ CD27^-^ |
| B-cells CD38 Dim | CD19^+^ CD38^Dim^ |
| Plasmablasts | CD19^+^ CD38^Bright^ CD27^+^ |
| Granulocytes | CD45^+^ SSC^high^ |
| Monocytes | CD45^+^ SSC^middle^ |
